# Supplementary material for: Dose estimates and their uncertainties for use in epidemiological studies of radiation-exposed populations in the Russian Southern Urals
Source: PLoS One. 2023 Aug 10;18(8):e0288479. doi: 10.1371/journal.pone.0288479 (PMC10414627; doi:10.1371/journal.pone.0288479)
Supplement: S1 File — The report provides details on the data and models used for the stochastic approaches in TRDS-2016MC. (PDF) [file pone.0288479.s001.pdf]

UNCERTAINTIES IN PARAMETERS FOR DOSE CALCULATION IN THE TECHA RIVER DOSIMETRY SYSTEM TRDS-2016MC

B.A. Napier, E.A. Shishkina, M.O. Degteva

Urals Research Center for Radiation Medicine
Chelyabinsk, Russian Federation

Pacific Northwest National Laboratory
Richland, Washington, USA

Unscheduled Report

US-Russian Joint Coordinating Committee on Radiation Effects Research
Project 1.1

"ENHANCEMENTS IN
THE TECHA RIVER DOSIMETRY SYSTEM"

April 2020 (edition of 2022)

TABLE OF CONTENTS

Abstract 1
1. Introduction...2
2. Basic equations for calculations of individual doses for the Techa River and EURT................3
3. Uncertainty of the TRDS equation parameters..5
  3.1. Parameter distributions for the Techa River pathways...6
    3.1.1. External exposure...6
    3.1.2. Internal exposure...15
  3.2. Parameter distributions for the East Urals Radioactive Trace (EURT) pathways............19
    3.2.1. External exposure...19
    3.2.2. Internal exposure...20
4 Parameter distributions for the atmospheric iodine pathways.......................................22
  4.1. Environmental Accumulation (DESCARTES) Equations...23
  4.2. Individual Dose (CiderF) Equations..26
  4.3. Definition of Parameters for DESCARTES and CiderF Equations................................27
  4.4. Release Source Terms...30
  4.5. Iodine Speciation 31
  4.6. Biosphere and Individual Parameter Uncertainty Distributions.................................32
  4.7. Environmental Accumulation Modelling 38
    4.7.1. Frost Date Libraries 38
    4.7.2. Animal Diet Libraries...39
    4.7.3. Commercial Leafy Vegetable and Milk Distribution 40
    4.7.4. Runs of the DESCARTES Code..41
    4.7.5. Individual Diets...41
5. Doses from medical exposure...42
6. Summary of TRDS parameter uncertainties...43
Acknowledgements...48
References...48

International Copyright © 2020 by the Urals Research Center for Radiation Medicine.
All rights reserved.

ABSTRACT

The Techa River Dosimetry System (TRDS-2016) has been designed to estimate radiation doses to individuals who were exposed while living in the vicinity of the Mayak Production Association. Individual dose estimates provided by complex dosimetry systems which are based on historical reconstructions are subject to considerable uncertainty.

The latest dosimetry system, TRDS-2016, includes a stochastic version to provide the individual dose uncertainties. In addition, doses to thyroid for most of the cohort are calculated using a variant of the Hanford Thyroid Dosimetry System (the CiderF code); this system has its own set of uncertain parameters.

Assignment of parameter uncertainty was performed based on the analysis of different sources of information on behavioral patterns, intakes, and environmental radionuclide deposition that allow formulating the parameters' uncertainty structures and selecting the parameter distributions in the TRDS-2016MC. Parameters were studied, specified and described in different reports over the past ten years.

This document pulls together all information on the current status of the TRDS-2016MC and CiderF. The assumptions to be revised are discussed. Thus, the specific purpose of this document is to describe the basis of the assumptions on the uncertainties in the parameters for calculation of doses due to residence in the Techa River basin in accordance with the data used in TRDS-2016D and CiderF.

1. Introduction

Population exposure in the Urals region occurred as a result of failures in the technological processes in the Mayak plutonium facility in the middle of the 20th century. A major source of environmental contamination was the discharge of about $1x10^{17}$ Bq of liquid waste into the Techa River in 1949–1956. Residents of many villages downstream from the site of release were exposed via a variety of pathways; the most significant were the usage of the river water as a source of drinking water, external gamma exposure due to proximity to bottom sediments and the shoreline, as well as consumption of contaminated foodstuffs.

It is known that the Urals population had additional sources of exposure. The most important was an explosion in the radioactive waste-storage facility in 1957 (the so-called Kyshtym accident) that formed the East Urals Radioactive Trace (EURT) due to dispersion of 7.4×10^{16} Bq into the atmosphere. Other sources of exposure include the gaseous aerosol releases from the Mayak facility in 1949–1957 – particularly those of ^{131}I - and windblown contamination from Lake Karachay, when this contaminated lake dried out in 1967.

The series of radioactive releases that occurred in the same region in different years and the intensive migration of the population within the contaminated area are specific features of the Urals situation. This determined the approach to epidemiological follow-up: selecting a fixed cohort and tracing all places of residence for each subject in the cohort since the beginning of radioactive contamination. The Techa River Cohort (TRC) and EURT Cohort (EURTC) include approximately 48,000 members and represent an unselected population consisting of two distinct ethnic groups. The members of the TRC and EURTC were exposed to chronic radiation over a wide range of doses, but at low-to-moderate-dose rates.

The combined dosimetric and epidemiologic study of these cohorts is deemed important, as this group is one of few that can be studied to examine the question of whether there is a dose-rate-reduction factor in the induction of stochastic effects by radiation. This question represents a central issue in radiation protection of workers and the public. The overall scientific hypothesis to be tested by the combined dosimetric and epidemiologic study of these groups is 'Radiation dose delivered at low dose rates is equally as effective (in causing cancer and other stochastic effects) as the same dose delivered at high dose rates'.

Russian and US scientists have been working together to perform dose reconstruction and epidemiologic follow-up for the TRC since 1995. The previous version of the Techa River Dosimetry System, TRDS-2009, was an update of the TRDS-2000 system. In order to provide more accurate and precise estimates of individual dose (and thus more precise estimates of radiation risk) for the members of the exposed cohorts, continuing improvements to TRDS-2009 have been made. The latest dosimetry system, TRDS-2016, consists of both deterministic (point estimate) and stochastic (including uncertainty) versions, which use the same basic input data but are coded separately. This provides the project with the opportunity for comparative analyses for quality assurance, as well as providing the end users of the dose estimates with various opportunities for analysis depending upon the requirements of the analysis.

This report describes the basic information used for stochastic approaches in TRDS-2016MC and the parallel atmospheric radioiodine code CiderF.

2. Basic equations for calculations of dose and uncertainty for the Techa and EURT

As has been described in our Milestones (Degteva et al. 2017a; 2017b; Napier et al. 2018a; Napier et al. 2018b) the method used for the TRDS-2016D dose calculations for exposure to radionuclides released into the Techa River and the EURT is relatively simple and can be written as a single equation consisting of four parts:

$$D_{o,Y,i} = \sum_{y=y_{min}}^{P\leq Y} \left[\sum_{L} M_{y,L,i} \begin{bmatrix} \left(\sum_{r} I^{*}_{y,r,L}(\tau_i)\, DF_{r,o,Y-y}(\tau_i) \right) + A_o\, D_{Riv,L,y} \left(T_1(\tau_i) + R_{\frac{out}{Riv},L} \left(T_2(\tau_i) + R_{\frac{in}{out}} T_3(\tau_i) \right) \right) + \\ G_{Sr,L}\, \delta_y \left\{ \sum_{r} E_{r,y}(\tau_i)\, DF_{r,o,Y-y}(\tau_i) + A_0\, D_{Sr,y} \left[(1 - T_3) + R_{\frac{in}{out}} T_3(\tau_i) \right] \right\} \end{bmatrix} \\ + \sum_{e_i} X_{o,i}(e_i, y, \tau_i) \\ + D_{AtmI,y,L,i} \right] \quad (1)$$

Here the upper line in the internal brackets represents the dose from the Techa River from internal exposures (left portion) and external exposures (right portion), the second line represents dose from exposure to fallout from the East Urals Radioactive Trace (EURT) from internal exposures (left portion) and external exposures (right portion), the third line represents dose from medical x-ray examinations (values of which have not been updated since 2013), and the fourth line represents doses from atmospheric iodine. Note that doses from atmospheric iodine from Mayak stack releases ($D_{AtmI,y,L,i}$) are included in the TRDS via a separate set of calculations described in Section 4 below; the annual dose to thyroid from the atmospheric releases is added to the other doses. The individual components are:

$D_{o,Y,I}$ = absorbed dose (Gy) in organ o accumulated through calendar year Y to individual i;

Y = the calculational endpoint for a particular individual (can vary according to the analyst's wishes within the range 1950–2015);

b_i = the year of birth of individual i;

y = year of environmental exposure (external irradiation and intake of nuclides). The minimum value of y in the summation is $y_{min} = MAX\{1950, b_i$, year of first moving to the Techa River or EURT area$\}$;

P = the endpoint of external exposure and intake of radionuclides for a particular individual (can vary within the range 1950 – Y, $P \leq Y$);

L = location (settlement) identifier;

$M_{y,L,i}$ = fraction of year y spent in location L by individual i;

r = identifier of ingested radionuclide (^{89}Sr, ^{90}Sr, ^{95}Zr, ^{95}Nb, ^{103}Ru, ^{106}Ru, ^{137}Cs, ^{141}Ce, ^{144}Ce or ^{131}I);

τ_i = $y - b_i$, the age of individual i in year y (years);

$I^*_{y,r,L}$ = intake function (Bq) for year y, radionuclide r, and location L (function of age τ, related to y);

$I^* = I \times \xi_i$, where ξ_i is a modifier predetermined for individual i equal to one (i.e., the village average), IMR_i (individual to model ratio), or HSR_i (household specific ratio), discussed below;

$DF_{r,o,Y-y}$ = conversion factor (Gy Bq^{-1}) for dose accumulated in organ o in year Y-y from intake of radionuclide r in year y (function of age, related to y, and gender if applicable); Y-y = time since intake, years;

A_o = conversion factor from absorbed dose in air to absorbed dose in organ o (function of age, related to y);

$D_{Riv,L,y}$ = absorbed dose in air near river shoreline at location L received in year y (Gy);

$R_{out/Riv,L}$ = ratio of dose rate in air outdoors at homes to the dose rate by the river at location L;

$R_{in/out}$ = ratio of dose rate in air indoors to that outdoors;

T_1 = time spent on river bank (relative to whole year) (function of age, related to y);

T_2 = time spent outdoors (relative to whole year) (function of age, related to y);

T3 = time spent indoors (relative to whole year) (function of age, related to y).

$G_{Sr,L}$ = surface deposition of ^{90}Sr (Bq m^{-2}) at location L from fallout from the EURT;

δ_y = 0 or 1 depending on y. For the EURT, $\delta_y = 0$ for $y < 1957$;

$E_{r,y}$ = intake function per unit surface deposition of ^{90}Sr (Bq per Bq m^{-2}) for EURT for year y, radionuclide r (function of age, related to y), further described below;

$D_{Sr,y}$ = absorbed dose in air (Gy) received in year y per unit surface deposition of ^{90}Sr (Bq m^{-2}) from fallout from the EURT;

$X_{o(e,y,\tau)}$ = absorbed dose to organ o (Gy) from medical examination e in year y for age τ; and

$D_{AtmI,y,L,i}$ = dose from atmospheric iodine in year y at location L to individual i. This particular dose is calculated in a separate set of computer programs as described by Napier et al. (2015), Eslinger and Napier (2013a; 2013b) and Napier et al (2018b). Details are provided in Section 3.3 below.

The intake function $I_{y,r,L}$ is a complex, time-dependent function derived from a combination of data from tooth beta counting and the whole-body counter (Tolstykh et al. 2011). The village-average intake function $I_{y,r,L}$ for each year y is calculated as:

$$I_{y,r,L}(\tau) = I^{Sr90}_{y,R} \times \alpha^{Sr90}_{\tau,R} \times f^{Sr90}_{L} \times R^{L}_{y,r/Sr} \tag{2}$$

where $I^{Sr-90}_{y,R}$ = annual ^{90}Sr intake for adult residents of the reference settlement (Muslyumovo) in year y;

$\alpha^{Sr-90}_{\tau,R}$ = annual ^{90}Sr intake for other age groups relative to that for adults living in the reference settlement;

f_L^{Sr-90} = annual ratio of ^{90}Sr intake for location L to ^{90}Sr intake for residents of the reference settlement; and

$R_{y,R/Sr}^L$ = annual ratio of radionuclide (r)-to-^{90}Sr intake for location L in year y.

Note that there is also a special intake function for ^{137}Cs that has slightly different application than the simple one described above. It replaces the $I^*_{y,r,L}$ intake function with a variant that includes additional considerations; cesium intake occurred via consumption of both river water and milk (Tolstykh et al. 2013).

The empirical distributions of dose estimates can be generated independently for separate individuals who share no common sources of uncertainty. However, when uncertain parameters used in the calculation of estimated doses are common to more than one individual (either correlated or shared), or when epistemic and aleatory uncertainties are both present, the empirical joint distributions of the correlated uncertainties should be generated. This can be accomplished by modularizing the Monte Carlo process, so that the Monte Carlo realizations of parameters that are shared among individuals can be preserved for repeated use. The first stage is to identify uncertain quantities that are a common to groups or all individuals in the cohort. For each of these quantities, subjective probability distributions are specified that reflect the state of knowledge about the true but unknown values. From these distributions, a Monte Carlo sample of alternative realizations is drawn. These alternative realizations of shared parameters may be saved in a static database.

The second stage involves use of Monte Carlo procedures to produce an alternative realization of the distributions of the unshared parameters for each individual. When combined with the vectors of shared parameters, the calculation results in the joint distributions of uncertain results for all members of the cohort. The number of alternative realizations of the dose is made large enough to enable a subjective confidence interval to be obtained. This two-step process is referred to as a two-dimensional Monte Carlo analysis (NCRP 2010). Both the TRDS-2016MC and CiderF codes use this approach.

The basis for approaches to TRDS parameter uncertainties is described in the next section.

3. Uncertainty of the TRDS equation parameters

Each of the parameters defined above has been studied over the course of Project 1.1 and is tabulated in the TRDS databases (Degteva et al. 2017a, b). In the basic equation, the parameters b_i, $y_{\min}$, P, $M_{y,L}$, and τ for each individual come from individual-life-history information and are a series of constants (although there is some uncertainty associated with move dates $M_{y,L}$). All of the other parameter values are either calculated or approximated and have associated uncertainty. The basis of the uncertainty definition for each of these parameters was discussed in several papers and reports and is summarized here.

3.1. The Techa River exposure situation

3.1.1. External exposure

Conversion factor from absorbed dose in air to absorbed dose in organ (A_o)

The conversion factor from absorbed dose in air to absorbed dose in organ o, Ao, is a mild function of radiation energy. However, there is a large plateau in the energy-dependent response between about 0.08 and 1.3 MeV (Eckerman and Ryman 1993; Petoussi et al. 1991), the energies of most interest for the radionuclides discharged to the Techa River. The factor A_0 has been assigned a combination of shared and unshared uncertainties (Type AB). For TRDS-2016MC calculations, this factor was assigned organ-specific shared uncertainties uniformly distributed between 0.9 and 1.1 to account for the potential minor variations in gamma energy for the environmentally-distributed sources. This factor was also assigned an unshared uncertainty multiplier of between 0.9 and 1.1 to account for individual differences in height and body mass.

More detailed overview of the sources of the overall uncertainty of A_o was performed analyzing the results of Monte-Carlo simulations of air kerma and organ-specific dose factors calculated for different soil element composition and densities, different depth of radionuclide deposition, phantoms of different age (Schwarz and Bolch (2014). The results were described in Addendum to Milestone Report 11 Part 1 (Degteva et al. 2016) and published in Shishkina et al. (2016).

A_0 calculated for ^{95}Zr, ^{95}Nb, ^{144}Ce/^{144}Pr/^{144m}Pr, ^{106}Ru/^{106}Rh, ^{91}Y were practically the same as those for ^{137}Cs/^{137m}Ba (the difference was ≤7%). It was also shown that the variations in chemical composition and possible values of soil density result in uncertainty that does not exceed 5%. Therefore, it has been confirmed that the shared component of uncertainties is about 10% and will not be changed in further calculations.

The uncertainty of A_o associated with the different depth of ^{137}Cs/^{137m}Ba deposition was accepted ad-maximum as 7%. The maximum non-excluded systematic error related to sex differences was estimated as 4%. Nevertheless, A_o is highly sensitive to the height of a phantom (which is age-specific). The dependence of this parameter on the height of computational phantoms (representing people of different age) was used for evaluation of the uncertainty due to individual variability of human body height. This variability was estimated from the anthropometric data stored at the URCRM (for the Urals rural population) except for 1year old children for whom the WHO data were analyzed (WHO 2006). The height of a human being is normally distributed withing a certain age group, and it results in the uncertainty of $A_o < 12\%$ (for all organs). Therefore, the overall unshared component of uncertainty s is better represented by a normal distribution with mean of 1.0 and standard deviation of 0.16 (conservative estimate of overall unshared uncertainties). This distribution will be used in future calculation runs.

Absorbed dose in the air near the river shoreline ($D_{Riv,L,y}$)

$D_{Riv,L,y}$ describes the dose rates in separate villages or village clusters, as derived from the Techa River transport model and source terms. Initially, this term was derived from the historical measurements of dose rate in each village. The current version of TRDS is based on the modeling of dose rates due to radioactive contamination of the shoreline predicted by the Techa River model of radionuclide transport (Shagina et al. 2012b). The uncertainty of model

predictions was estimated by comparison with measurement results taking into account measurement uncertainty (Degteva et al. 2016). A shared uncertainty within a village is given as a multiplier with mean 1.0 and standard deviation of 0.1.

The comparisons indicate that the modeled dose rates replicate well the measurements for distances of up to 200 km, being within about a factor of two (Figure 3.1). For the longer distances, the model results tend to underpredict the measurements, although these underpredictions are of small values.

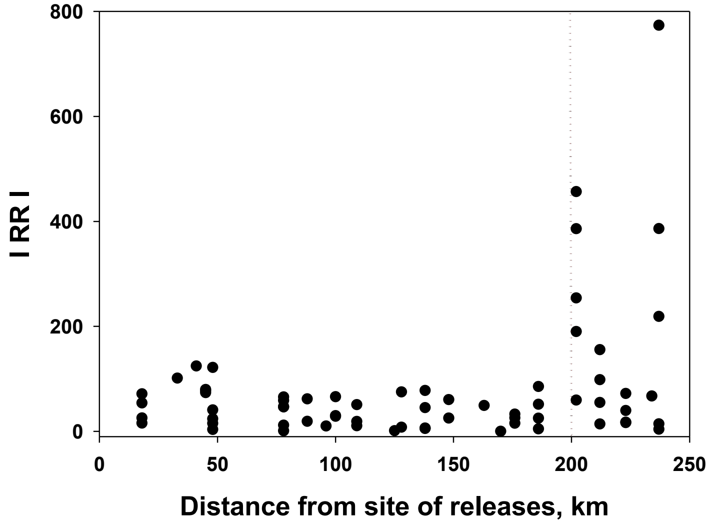

Figure 3.1. Distance dependence of the absolute values of relative residuals (|RR| in percent) (from Degteva et al. 2016).

The uncertainty of a measurement includes the uncertainty of repeatability due to equipment characteristics (about 20%) and due to the specifics of the environmental radionuclide geometry and local radionuclide distribution. The latter was evaluated based on the detailed gamma-radiation survey performed in Muslyumovo in 2005 (SOUL 2006). The measurements were performed with Dosimeter DRG-01T at a height of 1 m above ground/water at every node of a 5 m × 5 m grid within the 50 m × 55 m floodplain area. A total of 80 measurements, performed within 10 meters of the shoreline, were taken for analysis. The distribution of measurement results is well fitted by a lognormal function with GSD=1.77.

As a result of combination of all sources of uncertainties, village or village-cluster values of $D_{Riv,L,y}$ in the upper 200 km of the river will be assigned an uncertainty multiplier with a mean of 1.0 and a standard deviation of 0.4, truncated at 0.1 to avoid negative values. This will provide an overall uncertainty distribution approximating a range of about 0.5 to 2. Village or village-cluster values of $D_{Riv,L,y}$ in regions beyond 200 km from the source will be assigned a multiplier with a lognormal distribution with mean of 2.0, GSD of 3.31, truncated at 0.05 and 10. This will provide an overall 90% uncertainty range from 0.13 to 7, and adjust the underprediction bias.

The parameter $D_{Riv,L,y}$ requires consideration of seasonal changes due to shielding by a snow layer for winter months of each year. A snow shielding coefficient for winter months in both TRDS versions was taken to be 0.5 and applied to dose rates at the riverbanks and in residential

areas for the period November - March (Shagina et al. 2012a). These estimates were derived from the data of the Chelyabinsk Meteorological Office: the usual snow depth is 25-30 cm, the duration of the snow cover is about 150 days per year. According to weather monitoring performed in Ozyorsk in the 1950s, average snow depth was 32 cm, and average duration of snow cover was 140 days per year (Brokhovich 1996). With regard to snow shielding, climate change observed during the last 60 years is considered to be negligible.

A snow shielding coefficient for winter months was evaluated experimentally during field trips to Muslyumovo and Metlino, where dose rate measurements were performed at the same points in the Techa River floodplain in winter and in summer (SOUL 2006; 2007). Snow depth varied from 10 to 60 cm in Muslyumovo and from 20 to 100 cm in Metlino. The snow shielding coefficient varied for maximum and minimum depth of snow from 0.469 to 0.568 (mean = 0.53; CV=6%). Thus, the snow shielding coefficient assumed in the TRDS (0.5 with associated 10% uncertainty) appears reasonable.

<u>Outdoor-to-riverbank dose-rate ratios ($R^{L}_{out/riv}$)</u>

Approaches to evaluation of $R^{L}_{out/riv}$ in TRDS-2016MC were specific to the data on exposure-rate measurements available for a particular settlement. If measurements of the exposure rate with the distance from the shoreline in the 1950s were available for a village, these data were used to derive values of $R^{L}_{out/riv}$. Otherwise, the ratio was derived from data on survey of exposure rates performed in 1970 (Korsakov et al. 1970) and 1981–1983 (Lebedev et al. 1985), as was used in (Vorobiova et al. 2009). For locations where measurements of the decrease of exposure rate with the distance from the shoreline were absent, the ratio was evaluated from exposure-rate measurements performed in nearby villages with similar characteristics of the riverbank. Because the actual distances of individual residences from the river were largely known but not yet included in the databases, it was necessary to assume that specific individuals could live in any house. Therefore, the bank-to-residence and indoor-to-outdoor dose-rate ratios were treated as loguniform distributions between the lower and upper observed bounds within each village. These were held constant from year to year within a realization.

The parameter for a particular individual is primarily determined by the location of an individual household where he/she spent most of his/her time. Initial data and approaches to individualization of external doses were described in Degteva et al. (2011). A task of that report was to associate a particular household (and all its members) with a proper conventional area on the digital map of the Techa River settlement where these people were exposed. This task was performed for the upper Techa Riverside villages where significant levels of exposure rate were observed. According to information initially available, data for the residents of the upper Techa River settlements were grouped into clusters with homogeneous conditions of external exposure. Parameters under consideration included distance from the contaminated river shoreline and distance from the release site. For each cluster, the average outdoor-to-riverbank dose-rate ratio and its range were evaluated. The results of this task are given in Table 3.1. As can be seen, it was possible to implement clustering for 10 upper Techa River villages for which detailed maps

Table 3.1. Outdoor-to-Riverbank Ratios assumed for TRDS-2016 (according to Shagina et al. 2012).

Settlement	Distance from the site of releases, km	Average outdoor-to-riverbank ratio	Minimum – maximum range
Metlino	7	0.013	$1.1\cdot10^{-3}$-0.084
Cluster 1		0.042	0.036-0.045
Cluster 2		0.020	0.013-0.028
Cluster 3		0.059	0.046-0.075
Cluster 4		0.050	0.046-0.056
Cluster 5		0.015	$6.0\cdot10^{-3}$-0.037
Cluster 6		0.038	0.025-0.050
Cluster 7		0.023	0.020-0.031
Cluster 8		0.014	$9.7\cdot10^{-3}$-0.020
Cluster 9		$3.9\cdot10^{-3}$	$2.7\cdot10^{-3}$-$8.3\cdot10^{-3}$
Cluster 10		$7.6\cdot10^{-3}$	$3.4\cdot10^{-3}$-0.016
Cluster 11		$2.9\cdot10^{-3}$	$1.2\cdot10^{-3}$-$7.6\cdot10^{-3}$
Cluster 12		0.055	0.027-0.100
Cluster 13		$8.2\cdot10^{-3}$	$6.8\cdot10^{-3}$-0.010
Cluster 14		0.014	0.012-0.020
Cluster 15		$8.5\cdot10^{-3}$	$5.1\cdot10^{-3}$-0.012
Cluster 16		0.034	0.023-0.048
Cluster 17		$7.9\cdot10^{-3}$	$3.1\cdot10^{-3}$-0.026
Cluster 18		0.011	$4.4\cdot10^{-3}$-0.033
Cluster 19		$7.0\cdot10^{-3}$	$2.0\cdot10^{-3}$-0.025
Cluster 20		0.011	$8.5\cdot10^{-3}$-0.014
Cluster 21		0.028	0.013-0.053
Cluster 22		$6.1\cdot10^{-3}$	$1.2\cdot10^{-3}$-0.053
Cluster 23		0.015	$3.1\cdot10^{-3}$-0.100
Cluster 24		0.019	$2.4\cdot10^{-3}$-0.075
Techa Brod	18	0.060	$8.7\cdot10^{-3}$-0.33
Novoe Asanovo	30	0.055	$5.0\cdot10^{-4}$-0.19
Cluster 1		0.075	$5.5\cdot10^{-3}$-0.19
Cluster 2		0.037	$5.0\cdot10^{-4}$-0.061
Staroe Asanovo	33	$6.5\cdot10^{-3}$	$7.0\cdot10^{-4}$-0.0203
Cluster 1		$9.0\cdot10^{-3}$	$7.0\cdot10^{-4}$-0.02
Cluster 2		$2.8\cdot10^{-3}$	$1.6\cdot10^{-3}$-$4.3\cdot10^{-3}$
Nazarovo	35	0.015	$3.8\cdot10^{-3}$-0.031
Cluster 1		0.024	0.019-0.031
Cluster 2		$7.2\cdot10^{-3}$	$3.8\cdot10^{-3}$-0.012
Cluster 3		0.015	$3.8\cdot10^{-3}$-0.031
Asanovo [1]	33	0.028	$1.0\cdot10^{-3}$-0.094
M. Taskino	41	0.017	$1.9\cdot10^{-3}$-0.047
Cluster 1		$8.5\cdot10^{-3}$	$1.9\cdot10^{-3}$-0.013
Cluster 2		0.028	0.014-0.047

Settlement	Distance from the site of releases, km	Average outdoor-to-riverbank ratio	Minimum – maximum range
Cluster 3		0.017	$1.9\cdot10^{-3}$-0.047
Gerasimovka	43	0.044	$1.5\cdot10^{-3}$-0.21
GRP	45	0.018	$1.3\cdot10^{-3}$-0.098
Nadyrov Most	48	0.022	0-0.20
Cluster 1		0.076	0.066-0.085
Cluster 2		0.076	0.030-0.20
Cluster 3		$3.7\cdot10^{-3}$	$4.0\cdot10^{-4}$-0.022
Cluster 4		$7.0\cdot10^{-5}$	0-$3.0\cdot10^{-4}$
Nadyrovo	50	0.062	$4.7\cdot10^{-3}$-0.23
Cluster 1		0.10	0.040-0.22
Cluster 2		0.027	0.024-0.037
Cluster 3		0.10	0.043-0.23
Ibragimovo	54	0.17	0.018-0.54
Cluster 1		0.30	0.12-0.54
Cluster 2		0.067	0.018-0.094
Isaevo	60	0.10	$1.0\cdot10^{-3}$-0.48
Cluster 1		0.24	0.084-0.48
Cluster 2		0.054	$1.5\cdot10^{-3}$-0.13
Cluster 3		$1.4\cdot10^{-3}$	$1.0\cdot10^{-3}$-$4.0\cdot10^{-3}$
Cluster 4		0.10	$1.0\cdot10^{-3}$-0.48
Podssobnoe hoz.	70	0.031	0-0.23
Muslyumovo station	75	0	0-0.14
Muslyumovo village	78	0.016	0-0.74
Kurmanovo	88	0.048	0-1.7
Karpino	96	0.033	$5.0\cdot10^{-4}$-0.54
Vetrodujka	100	0.048	$2.0\cdot10^{-3}$ – 0.43
Zamanikha	105	0.072	0.03-0.40
Brodokalmak	109	$6.0\cdot10^{-3}$	0-0.97
Osolodka	125	$9.8\cdot10^{-4}$	0-0.97
Panovo	128	$7.1\cdot10^{-3}$	0-0.74
Cherepanovo	137	$6.2\cdot10^{-4}$	0-1.15
Russkaya Techa	138	0.029	$7.5\cdot10^{-3}$ – 0.098
Baklanovo	141	0.025	$5.0\cdot10^{-4}$-0.39
Nizhnepetropavlovskoye	148	$3.0\cdot10^{-3}$	0-1.1
Beloyarka-2	155	$2.2\cdot10^{-3}$	0-1.1
Lobanovo	163	$7.7\cdot10^{-4}$	0-1.1
Anchugovo	173	$1.5\cdot10^{-3}$	0-0.75
Verkhnyaya Techa	176	$8.4\cdot10^{-5}$	0–0.068
Skilyagino	183	0.044	0-1.2
Bugaevo	186	0.039	0.034-0.7
Dubasovo	200	0.039	0.034-0.7

Settlement	Distance from the site of releases, km	Average outdoor-to-riverbank ratio	Minimum – maximum range
Bisserovo	202	$8.2 \cdot 10^{-3}$	$2.8 \cdot 10^{-5}$-1.1
Shutikhinskoye	202	0	0-0.46
Progress	207	$8.2 \cdot 10^{-3}$	$2.8 \cdot 10^{-5}$-1.1
Pershinskoye	212	$1.0 \cdot 10^{-4}$	0-0.01
Klyuchevskoye	223	0.19	0.14-3.5
Markovo	230	$2.4 \cdot 10^{-3}$	0-1.7
Ganino	234	$6.9 \cdot 10^{-4}$	0-0.72
Zatechenskoye	237	0.18	0.14-3.6

[1] Includes Novoe Asanovo, Staroe Asanovo and Nazarovo.

were available. Only crude schemes of residential areas were available for the remaining three villages (Gerasimovka, GRP, and Podssobnoe hoz.), that did not allow grouping into clusters. Central (average) estimates are used for deterministic dose calculations in TRDS-2016D. For stochastic dose estimates log-uniform distributions are assumed between the lower and upper bounds, unless the individual has a known address in one of the village clusters, in which case the cluster value is used with a uniform multiplier btween 0.5 and 1.5.

Behavioral pattern parameters (T_1, T_2, T_3)

Model behavior factors T_1, T_2, T_3 – the fraction of time spent in different locations for different age groups – were derived from observational data (summarized in Vorobiova et al. 1999for Musluumovo and Brodokalmak in the 1990s (Balonov et al. 2007; Kravtsova et al. 1994) and on the Tom' River in Siberia in the 1970s (Maslyuk 1980)) of typical life-style patterns for different age groups of Techa Riverside residents. Table 3.2 exemplifies the data on behavior factors for different age groups available. The terms T_1, T_2, and T_3 were originally assigned generic values, depending on the age of the individual in year y. These times were assumed to vary by up to 30% for individuals, constrained to the total hr/year. They were allowed to change from year to year to account for individual circumstances.

Table 3.2. Typical life patterns for different age groups of the Techa Riverside residents.

Age group, years	Period of time spent at specified site, hours per year			
	Shoreline (summer time)	Residence area (outdoors)	Residence area (indoors)	Far from the river (uncontaminated territory)
<7	45	2235	6480	0
7–15	150	2130	5760	720
16–59	150	1410	3960	3240
≥60	150	2490	6120	0

It was discovered that the "30% variation" was actually relative errors of mean of these measurements, and the original uncertainty ranges were unrealistically narrow. For TRDS-2016MC, the range on the parameters was revised so that each of T_1, T_2, T_3 was multiplied by a lognormal distribution with a mean of 1.0 and GSD of 2.7 to more closely align with the original observations. The three time periods were then summed with the time far from the river (T_4) and the total then normalized to 8760 hours/year.

Planned Improvements in behavioral pattern parameters:

A detailed analysis of the data available was performed in order to re-evaluate the distribution of time spent near the Techa River (parameter T_1 which is important for external dose assessment). Re-evaluation was done because the data on critical group of people are not representative. The lifestyle of people in the 1990s may be different from that in the 1950s. Moreover, according to Marey et al. (1961), the "critical group" is a group of residents of a certain settlement whose external exposure doses are about 3 times higher than the population-average doses in this settlement. Critical groups represented 10-30% of the population. Higher external exposure of an individual can be mainly due to the fact that he/she spent much more time at the riverbank than a typical resident did. The value of 20% was assigned as the population fraction spending more time at the riverbank than other people.

Another reason for T_1 re-evaluation is as follows: some people did not spend time at the riverbank at all. This fraction of Techa riverside residents was estimated on the basis of the survey data on the river use. Survey data of 416 Metlino residents on the use of the river, which were conducted in 1953-1955, were derived from the URCRM archive. It should be noted, the surveys were conducted in a free form (not formalized). Only 47% of people were asked about swimming. About 80% of the people were asked if they accessed the river. Additional sources of information were the survey data on how residents used the river and floodplain (e.g., whether they swam, went fishing, washed clothes, made hay on the floodplain) in the 1950s. These surveys were conducted in 1970s and covered about 2000 people from different settlements along the Techa River. Only surveys of people born before 1946 were considered in the analysis. Table 3.3 provides a comparison of the fractions of people who didn't use the river according to survey data.

Table 3.3. Percentages of people who visited the river

Year of the Survey	N	Didn't swim	Didn't visit the river
Metlino			
1953-1955	197 answers about swimming and 324 answers about the use of the river water	23%	15%
1970s	126	17%	12%
Whole river			
1970s	1698	16%	10%

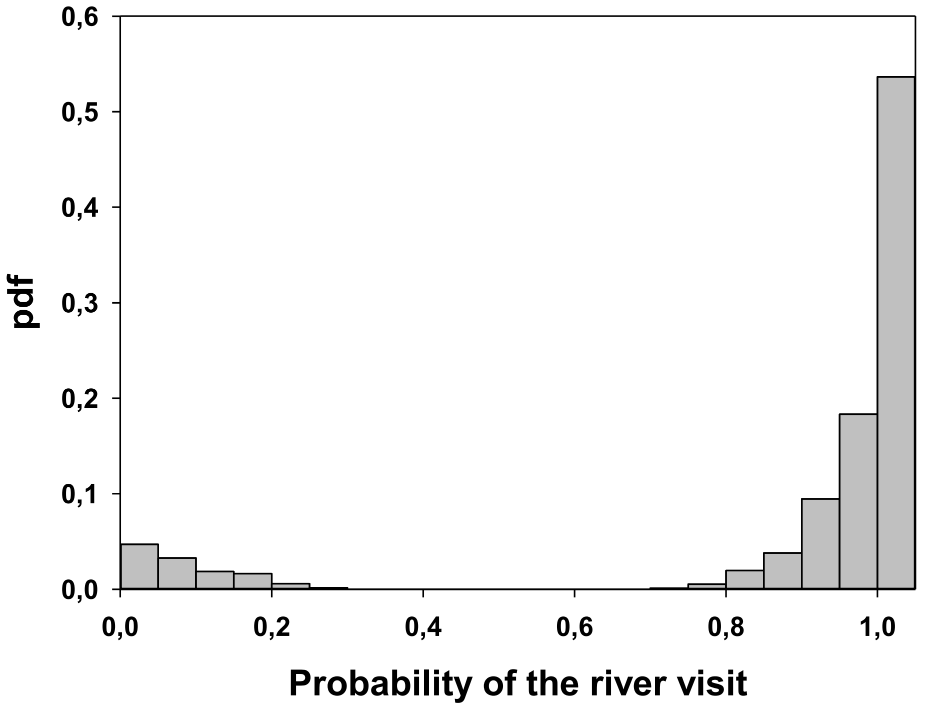

Figure 3.2. Probability density to describe the frequencies of people who visit the Techa River with different probability.

According to the survey conducted 20 years after the period of interest, the estimate of fractions of individuals who swam as well as who used the river did not differ significantly from the results obtained in 1953-1955. Therefore, we assume that about 13% (weighted average) of the Techa riverside residents didn't visit the river bank. A probability density function for river visiting has been constructed by convolution of the river visit probabilities (13% - did not visit and 87% - visited) perturbed randomly by right-side Norm(0;0.1) for non-visitors and left-side Norm(0;0.1) for visitors (Figure 3.2).

The probability obtained was multiplied by the probability of time spent at the riverbank typical of the general population. According to the study on the time spent at a river bank for residents of the Tom River performed in 1973-1977 (Sayapina et al. 1977) that we have recently come across, an average annual value of time spent at the river bank for people aged $\geq$7 years is 0.66 hours per day (main group of people – 67%), which also agrees with the similar study of Maslyuk (1980) on the Tom' River in Siberia in the 1970s. According to Sayapina et al. (1977), the critical group (20% of the people) spent 1.48 times longer time at the riverbank than other people. We assume a lognormal shape for the distribution of individual variability in time spent at the riverbank with distribution width equal to 63% in terms of CV (Sayapina et al. 1977).

Taking into account the fact that the exposure rates at the river shoreline and in residential areas have been calculated on a monthly-basis in 1950-1951, seasonal changes in behavioral regimes should be also considered. According to Balonov et al. (2007) the time spent at the river shoreline in winter made up 30% of that in summer.

Convolution of all these sources of uncertainties allows obtaining the distribution function of T_1 parameter. Figure 3.3 shows the example of such a convolution for mean $T_1=1$ (≥ 7 years) in summertime.

The coefficient of variation for T_1 distributed as shown in Fig. 3 is 110%. Table 3.4 shows mean life-style patterns for different age groups of general population of the Techa riverside territory.

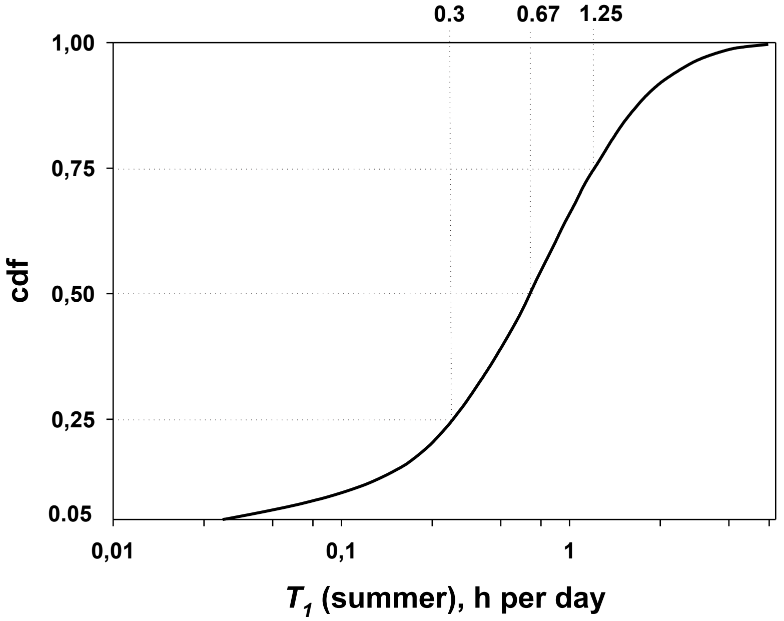

Figure 3.3 Example of cumulative distribution of T_1 in summertime for people aged ≥ 7 years.

Table 3.4. Mean life-style patterns for different age groups derived from observational data (Saurov et al. 1968, Saurov et al. 1992, Balonov et al. 2007, Maslyuk 1980, Sayapina et al.1977)

Age group, years	Period of time spent at specified site, hours per day							
	Shoreline (parameter T_1)		Outdoors, residence area (parameter T_2)		Indoors, residence area (parameter T_3)		Far from the river (uncontaminated territory T_4)	
	summer	winter	summer	winter	summer	winter	summer	winter
<7	0.5	0	6	6	17.5	18	0	0
7 – 15	1	0.3	6	6	15	17.7	2	0
16 – 59	1	0.3	5	6	10	10.7	8	8
≥60	1	0.3	6	6	17	17.7	0	0

Mean indoor and outdoor exposure time parameters (T_2 and T_3) were derived from the study of indoor population exposure performed in 1953–1955 (Saurov 1968) and from presentation of the survey on life-style patterns of residents of the Techa River settlements by Saurov (1992). However, no primary data on the variability of T_2 , T_3 as well as T_4 (staying far from the river due to traveling, working in the distanced places and so on) are available. The simplest assumption of uniform distribution with 30% of CV was used as a first approximation (Napier et al. 2001).

Further analysis of the sensitivity showed that the model is insensitive to variations in these parameters (Napier et al. 2001). Therefore, further refinement and data retrieval for specification of these parameters are not required.

The final implementation of the revised riverbank exposure parameters has not been finalized; however, it will require at least two unshared parameters (user/non-user, time) for each age group. These will be autocorrelated by year so that behavior is similar throughout life for each person.

Indoor dose rate reduction factor ($R_{in/out}$.)

The dose rate reduction factor $R_{in/out}$ for building occupancy was defined based on the analysis of archive data on dose rate measurements performed in 1981–1984. These data were described in the Appendix to Vorobiova et al. (1999). Analysis of the sensitivity showed that the model is insensitive to variation in $R_{in/out}$ (Napier et al. 2001). Therefore, further refinement and data retrieval for specification of the parameter are not required. The uniform distribution averaging 0.45 and ranging from 0.125 to 0.775 is retained for TRDS-2016 calculations. The parameter is assumed to be unshared to allow individual residence conditions to vary.

3.1.2. Internal exposure

A key parameter in determining internal dose is the average-intake function, $I_{y,r,L}$, of radionuclide r in year y at location L. The intake function $I_{y,r,L}$ is a complex, time-dependent function (Eq. 2) derived from a combination of data from tooth beta counting and the whole-body counter.

Reference function of annual ^{90}Sr intake ($I_{y,R}^{Sr-90}$)

Reference intakes of ^{90}Sr were fixed as a function of time for adult residents of Muslyumovo. The Muslyumovo reference function is generic for most other villages except Metlino. Metlino was the closest village to the site of radioactive releases (7 km), and the use of the Techa River as a source of water supply was prohibited in mid-August 1951. This is in contrast to all other villages, where wells to replace river water for consumption were not constructed until 1953–1954. Even before 1951, the main source of water supply of Metlino was not the Techa River but Metlinsky Pond on the Techa River, wells, and artesian wells. Moreover, the use of floodplain grass for fodder was insignificant in Metlino and the influence of the flood in spring 1951 that resulted in dilution of contaminated river water was less in Metlino than in Muslyumovo. Therefore the intakes in Metlino were evaluated separately.

The ^{90}Sr intakes in Muslyumovo during 1950 –1955 were reconstructed based on the data on ^{90}Sr measurements in teeth (1,268 measurements for 690 persons) (Kozheurov et al. 1994, 2000; Tolstykh et al. 2003) and 189 WBC measurements for 50 permanent residents of Muslyumovo born in 1920 –1926 (adults at intake) (Tolstykh et al. 2011). For the period 1954 –1980, the measurements of ^{90}Sr concentration in cow's milk from individual households in Muslyumovo were used (Tolstykh et al. 2011). The sources of information were as follows: 1) 150 measurements performed in 1956-1962 (Borovinskikh et al. 1963); 2); 575 measurements

performed at URCRM in 1968 - 1980 (Panteleev et al. 1971; URCRM database “Environment”), and water and milk consumption rates for adults and children (Tolstykh et al. 2011).

For Metlino, supplementary data on the total-beta activity in excreta (urine and feces) were used (Tolstykh et al. 2011): 260 measurements were performed during expeditions of the Institute of Biophysics in 1951–1952 (Marey et al. 1952); 2,420 measurements were available for period of 1952 - 1956 (Khokhryakov et al. 1968). Validation of dietary intakes was done using post mortem measurements of the total-beta activity in bone samples from Metlino residents since February 1952 (25 individuals) and in vivo WBC measurements of 159 individuals of similar years of birth.

The significant revisions to the intake function by Tolstykh et al. (2011), accounting for a large number updates and other modifications, resulted in year-by-year changes from earlier estimates of about 25% for most years, with the overall intake (the area under the curve) changing by less than this amount. It is believed that future revisions will, if anything, be smaller than these. Therefore, a shared uncertainty on the reference intakes represented by the generic curve is subjectively set to about 25%; thus a multiplier with a normal distribution of mean 1 and standard deviation 0.25 is used, truncated on the lower side at 0.05 to avoid negative values. A similar unshared multiplier is also used for each time period, strongly autocorrelated.

<u>Ratios of ^{90}Sr intake for children to intake for adults</u> ($\alpha_{\tau,R}^{Sr-90}$)

Annual ^{90}Sr intake for other age groups relative to that for adults living in the reference settlement was estimated (as described in Tolstykh et al. 2011) from the data on contribution of different foodstuffs to the dietary intake of ^{90}Sr:

1) 1950 –1951 study of adult residents of Muslyumovo (technical reports of the Biophysics Institute (Moscow)) and URCRM questionnaires about this period.
2) 1960 data on seasonal foodstuff consumption and analyses of duplicate provisions for adults and rough estimates of consumption for children of different ages (Skryabin 1971);
3) 1966 study of milk consumption from birth to 1 y old (Peremyslova 1967);
4) 1957 – 1962 study of daily consumption rates of milk and milk products for the Techa riverside residents using questioning and duplicated daily diet (Borovinskih et al. 1958; Marey et al. 1961, 1966; Borovinskih et al. 1963).
5) 1961 – 1963 study of daily consumption rates of milk and milk products for the rural population of Chelyabinsk Oblast (Skryabin 1971)

The data on daily diet and coefficients of ^{90}Sr transfer from river water to foodstuffs were taken from technical reports of the Biophysics Institute (Moscow) and from URCRM investigations of the Techa River residents.

The reference intake-functions were derived by normalization to average ^{90}Sr-body burdens in reference groups of adult permanent residents of Muslyumovo and Metlino (Tolstykh et al. 2011). It is assumed that the derivation of these correction factors on average has a shared normal uncertainty distribution with a standard deviation of about 10% (i.e., the tabulated value is within about 10% of the “true” average), and that individuals can also vary about the average by an additional unshared 20% (a multiplier of 1 with a standard deviation of 0.2, truncated at 0.01).

Annual ratio of ^{90}Sr intake for location L to ^{90}Sr intake for residents of the reference settlement (f_L^{Sr-90})

The assessment of f_L^{Sr-90} is based on the assumption that the ratio between ^{90}Sr intake in a particular settlement and the reference ^{90}Sr intake is equal to the ratio between the average ^{90}Sr-body burden for the particular settlement and the corresponding value for the reference settlement (Tolstykh et al. 2002; 2003). Because more than 95% of total-^{90}Sr intake occurred in 1950–1953, the average of normalized ^{90}Sr-body burdens in residents who lived continuously in a single Techa River village during at least the period of 1950–1953 were used for estimation of village-specific intake functions; the variability for unmeasured individuals was based upon the cumulative frequency distribution of IMRs within the individual villages.

An individual's measurements of body burdens of ^{90}Sr (with WBC) are used if they are available and appropriate, if not but the individual has measured relatives in the same household, an average is taken of those, or if neither are available, then the village average is used. The relation of the actual measurements to the model predictions is described using Individual-to-Model Ratios (*IMR*) (Degteva et al. 1999). IMRs are individually calculated on the basis of WBC measurements; to allow for measurement variability, model uncertainty, and other factors an unshared multiplier is estimated for every IMR as an input.

Table 3.5 gives statistical data on WBC measurements for residents of the Techa Riverside settlements (Shagina et al. 2007).

Table 3.5. Statistics on ^{90}Sr measurements of the residents of the Techa riverside settlements with the use of WBC-9.1.

Sample*	Number of persons	Number of measurements
WBC database and autopsy measurements	16,610	33,478
Persons born before 1950 and who lived on the Techa River for any time period (formal definition of the ETRC):	9,693	23,436
Residents with single or repeated WBC measurements > RDL**	5,526	16,994
Residents with a single WBC measurements < RDL**	2,801	2,801
Residents with repeated WBC measurements < RDL**	1,366	3,641

*Residents who arrived in the Techa River settlements after 1960 are excluded.
**RDL – reliable detection limit – the measurement threshold (which is dependent on both equipment performance and the body burdens due to global fallouts and natural ^{40}K) above which the WBC result is exceeding the background level with 95% of probability. The RDL measurements can be treated directly as individual measurements of ^{90}Sr due to radionuclide intakes from the Techa River.

The annual ratio of nuclide-to-^{90}Sr intake ($R^{L}_{y,R/Sr}$)

Intakes of other radionuclides (^{89}Sr; ^{141,144}Ce; ^{95}Zr; ^{95}Nb, ^{137}Cs, ^{103,106}Ru) due to residency in Techa River villages were obtained from the result of the research into the radionuclide source term and Techa River modeling (Shagina et al. 2012b). For short-lived radionuclides, the intakes are proportional to the estimated concentrations of these radionuclides in river water. Source term estimates have been made by several teams. Radioactive discharges into the Techa River were evaluated on the basis of radioecological monitoring and dosimetric modeling data in a joint project supported by the International Science and Technology Center (ISTC). The research team included staff of the URCRM, the Mayak Production Association, the Russian Federal Nuclear Center–All Russia Scientific Research Institute of Technical Physics (RFNC–VNIITF), the Institute of Plant and Animal Ecology of the Urals Division of the Russian Academy of Sciences, and the U.S. collaborators of Project 1.1, plus Dr. Owen Hoffman of SENES, Oak Ridge. Summary and analysis of the results of ISTC Project No.2841 on Reconstruction of the Techa River source term were presented in Degteva et al. (2008). The interpretation differed for some radionuclides by up to factors of two from those of Glagolenko et al. (2006; 2008). Thus, uncertainties on the quantities released into the Techa River are assumed to be within factors of two (a lognormal distribution with a mean of 1.0 with a GSD set such that the 90% confidence interval is from 0.5 to 2 times the median). This is applied independently to each radionuclide and held constant throughout the entire environmental realization. In addition, an individual village uncertainty for the river transport is superimposed by radionuclide as a uniform distribution between 0.5 and 1.5.

Intake of ^{137}Cs as a function of calendar year by adult residents of the villages contains two functions: (1) ^{137}Cs intake with river water and (2) ^{137}Cs intake with cows' milk. Therefore, two ratios are calculated for each village: (1) ratio of river water intake as a function of age for children to that for adults, and (2) ratio of milk intake as function of age for children to that for adults. The total dietary intake of ^{137}Cs, which mostly determined the levels of internal exposure of the soft tissues, is modeled with cow's milk as the main source of this radionuclide in the local diet after May 1951 (Tolstykh 2008; 2013). The algorithm developed by Tolstykh has shared components (initially by month, later by year) for water and milk intake as functions of age; these are assumed to have a normal uncertainty of 10%. It also has unshared components; these are assigned a normal uncertainty of either 10% for water intake or 20% for milk intake.

Dose conversion factors per unit intake ($DF_{r,o,Y\text{-}y}$)

Dose-conversion factors, $DF_{r,o,Y\text{-}y}$, are calculated using biokinetic models, and their uncertainties are determined mainly by the variability of metabolic parameters (Shagina et al. 2000). However, for ^{90}Sr, the individual variability in uptake and metabolism is actually captured in the *IMR* values, because the *IMR*'s reflect not only intake but also long-term retention. The remaining uncertainties in the dosimetric model are embodied within the specific effective energy quantity and are associated mainly with variations in masses, shapes and locations of the organ and tissue of the human body and with oversimplifications of the representations of certain complex anatomical structures in the body when calculating the energy deposition (NCRP 1998). Thus, the uncertainty in the dose-conversion component for ^{90}Sr is relatively low. The uncertainties in the dose-conversion factors for other radionuclides are larger, reflecting the lack of available measurements and the potential for individual variations in

uptake and retention. Because individual variations in uptake and retention will vary less from year to year than the variation among individuals, the dose-factor variability is held constant from year to year for a single realization of the dose estimate and only varied for additional realizations.

To date, the shared uncertainties for all organs (individually) for all radionuclides has been assigned multipliers with a mean of 1.0 and a standard deviation of 0.1, to indicate the relative centrality of the ICRP standard models. The unshared uncertainty for ^{90}Sr has been assigned a lognormal multiplier with mean of 1.0 and GSD of 1.25; the unshared uncertainties for all other radionuclides for all organs (individually) use lognormal multipliers with mean of 1.0 and GSD of 2. The strontium isotope distributions are currently being investigated as a task in Project 1.1 and the unshared individual uncertainties will be derived as functions of age and sex from that effort. Because increased deposition in one organ means potential decreases in others, a weak negative correlation between organs is applied

3.2. Dose due to exposure from the East Urals Radioactive Trace (EURT)

3.2.1. External exposure

Deposition of ^{90}Sr at location *L* for EURT fallout (G_{Sr_L})

During the first years after the accident, a great amount of work surveying the EURT structure was done by specialists of the Mayak PA Central Plant Laboratory, Ozyorsk, together with some other institutions, primarily with the Institute of Applied Geophysics, Moscow, and the Institute of Radiation Hygiene, St. Petersburg (JNREG 1997; Avramenko et al. 1997, Romanov et al. 1997 , Izrael 2000, Khokhryakov et al. 2002). Since 1958 Urals Research Center for Radiation Medicine (URCRM, Chelyabinsk) provides the monitoring of the population and environmental contamination of the territories of EURT. Territory contamination was described in terms of ^{90}Sr deposition taking into account the proportion of different radionuclide deposition. A retrospective map of ^{90}Sr deposition in the EURT territory in 1957 was developed by Izrael (2013). The data are collected in the URCRM database (Kostyuchenko et al. 2012; Molchanova et al. 2014).

TRDS-2016 databases (Tolstykh et al. 2006) include deposition densities for 84 EURT settlements located in the contaminated area with initial strontium depositions ≥0.1 Ci km^{-2}. Three of them (Berdyanish, Satlykovo, and Galikaeva) located in the most contaminated EURT area (480 Ci km^{-2} of ^{90}Sr deposition) were evacuated 7-14 days after the accident; 19 settlements (> 2 Ci km^{-2}) were evacuated in 1958 – 1959 years; 3 more settlements were evacuated in 1960 - 1974.

The uncertainty in the deposition averaged over a village is relatively small as a result of the extensive measurement campaigns. The shared component is given a unit multiplier with a normal distribution with a standard deviation of 0.05. However, intra-village variation is somewhat larger; an unshared variation within a resident village is assigned a unit multiplier with a normal distribution with a standard deviation of 0.2.

Additional non-uniformity of radionuclide contamination along the territory of a settlement is additionally accounted for in the intake description (internal exposure) and in the normalized dose rate in the air outdoors (external exposure), described below.

Normalized dose rate in air outdoors in year *y* from EURT fallout ($D_{Sr,y}$)

Time dependences of exposure parameters are normalized per 1 Ci km^{-2} of ^{90}Sr deposition in 1957, and thus, these data provide the basis for dose reconstruction using the classical 'deposition density-to-dose-conversion-factor' approach. In general, $D_{Sr,y}$ were calculated the same way as the absorbed dose in the air near the river shoreline ($D_{Riv,L,y}$). The difference was in the area and depth of contaminated soil. The uniformly contaminated infinite surface was assumed to be the source of exposure from the initial period up to November 1957 (first two months after the incident). The next period (up to 1962) 1 cm depth was assumed. Since 1962 the depth of ^{90}Sr contamination was assumed as equal to 5 cm.

As noted above, the dose rates for a given time period following a unit deposition decrease rapidly because of the decay of the shorter-lived radionuclides. A table of dose rates per month for 1957 through 1958, and per year from 1959 through 2015, is read. A shared uncertainty for each of the first 15 months is assigned a multiplier with a uniform distribution between 0.9 and 1.1; a final multiplier with the same range is used for all subsequent time periods because the chief determinant at longer times is ^{137}Cs. Individual unshared uncertainties are assumed to be related to the village inhomogeneity accounted for with G_{Sr_L}.

The influence of vertical distribution of radionuclides was mentioned in Shishkina et al. (2016). For example, $D_{Sr,y}$ calculated for surface radionuclide deposition differs from $D_{Sr,y}$ calculated for 1 cm of soil contamination by a factor of 2. The product of $D_{Sr,y}$ and A_0 (the same parameter as for the Techa River) reduces this difference (it becomes insignificant for depths of 1 cm and more. However, it still significant for the case of surface radionuclide deposition and 1 cm of soil contamination (the difference of $D_{Sr,y} * A_0$ is about 40%). In the future this should be also taken into account.

Behavioral pattern parameters (T_3)

These parameters are the same as for the Techa River. Only the term T_3 are used to represent times inside and outside buildings; the complimentory fraction (1- T_3) is used to represent terms inside buildings. In one sense, the lifestyle described is identical to that of a Techa River resident who does not visit the river.

Note that to date, slightly different values of the outdoor fraction (equivalent to T_2) have been used for the atmospheric radioiodine calcuations. Becuase external dose from the atmospheric iodine pathway is minimal, this difference is not critical, but could be harmonized in the future.

3.2.2. Internal exposure

The dose conversion factors per unit of intake used for the EURT are the same as for the Techa River. Therefore, the focus of this section is on internal exposure.

Normalized intake function for radionuclide *r* at location *L* ($E_{r,y}$)

In a manner similar to ingestion on the Techa River, intake functions have been developed for exposures to the EURT fallout. The basic approach considered by Tolstykh et al. (2017) for

the reconstruction of internal doses employ conversion factors $E_{r,y}$, that is, dose per unit ground deposition (Gy per kBq m^{-2}). The approach is based upon measurements of radionuclides in local foodstuffs and humans. The following information was used to develop the intake function:

1. Information on biological availability of the main radionuclides (Müller et al. 1999, Teverovsky and Ternovsky 1985, Annenkov et al. 1973, ICRP-67, Shagina et al. 2015)
2. Information of URCRM database on contamination monitoring in the EURT (Tolstykh et al. 2017).
3. Data on diet composition from the Statistics department of the Chelyabinsk Region (1975– 2011) and from Skryabin et al. (1971; 1985).
4. Measurements of β-activity in feces, WBC measurements and radiochemical measurements of post mortem autopsy (Tolstykh et al. 2017).

Table 3.6 describes the information available on EURT environmental monitoring. Table 3.7 shows the quantity of the data on individual radionuclide measurements.

Table 3.6. Summary statistics of information of URCRM database on EURT monitoring

Subject of monitoring	No. of measurements	Period of measurements
Environmental data		
Soil<br>Grass	6812<br>3830	1958 - 2011
Foodstuff		
Water supply<br>Bread<br>Milk<br>Potato<br>Vegetables<br>Meat	50<br>460<br>6840<br>822<br>430<br>45	1960 – 2011<br>1958 – 1982<br>1958 – 2011<br>1958 – 2011<br>1958 – 2011<br>1962 - 1977

Table 3.7. Summary statistics on available measurements of β-activity in feces, WBC measurements and radiochemical measurements of postmortem autopsy.

Subject of the study	No. of measurements	Period of measurements
Bone samples<br>WBC *in-vivo*<br>Feces samples	2170 (from 1646 persons)<br>369<br>226	1951 – 1989<br>1974 – 1997<br>1958 - 1959

The parameter derived has a wide uncertainty range in part because of the variability of the initial deposition within each village and in part because of the relatively small number of directly-applicable measurements. The parameter has an uncertainty distribution shared by radionuclide within a village with a lognormal multiplier with mean of 1.0 and GSD of 3. An unshared distribution to account for individual variability, by location (varies if the person moves from one EURT location to another), has been assigned a lognormal multiplier with mean of 1.0 and GSD of 2.

4. Parameter Distributions for the Atmospheric Iodine Pathway

The general approach to calculating individual doses to members of the public from historical releases of airborne 131I has the following general steps:

- Construct estimates of releases ^{131}I to the air from production facilities.
- Model the transport of ^{131}I in the air and subsequent deposition on the ground and vegetation.
- Model the accumulation of ^{131}I in soil, water and food products (environmental media).
- Calculate the dose to individuals by matching the appropriate lifestyle and consumption data for an individual to the concentrations of ^{131}I in environmental media at their residence location.

A number of computer codes were developed or adapted to model the transport and environmental accumulation of ^{131}I as part of the Hanford Environmental Dose Reconstruction Project. The equation set and associated parameters specific to Hanford are documented in (Snyder et al. 1994). The air transport code named HYSPLIT (Draxler et al. 2018) is being used to model the movement of ^{131}I in the air because it has access to a global database of meteorological information. The environmental accumulation code DESCARTES (Dynamic Estimates of Concentrations and Accumulated Radionuclides in Terrestrial Environments) has a published user's guide (Miley et al. 1994). An associated computer code for estimating annual doses to humans named CiderF (Calculation of Individual Doses from Environmental Radionuclides - Fortran) (Eslinger and Napier 2013a) was developed for this project from the HEDR CIDER code (Eslinger et al. 1994).

The first codes in this sequence (through DESCARTES) are used to produce the concentrations of ^{131}I in environmental media that a human will interact with. Once the DESCARTES code has finished, the individual dose code CiderF can be run multiple times for different individuals without rerunning the previous codes. In this manner, the parameters used in the source, environmental transport, and environmental accumulation steps are all shared with all subjects. The parameters used in the individual exposure and dose calculations are primarily unshared (although a few may be shared as needed).

Some leafy vegetable and milk products consumed by individuals are not grown or produced at the location where potentially exposed individuals live. In addition, some of these products are consumed long after they are produced, thus there is time for radioactive decay to significantly reduce the ^{131}I concentration in these foods. The calculation sequence implements an optional commercial distribution system for leafy vegetables and milk products. This allows an individual to eat leafy vegetables produced on a farm grown at a different location from their residence location. In addition, a two-step milk product distribution system is implemented. First, a dairy farm produces milk (the dairy farm is called a creamery in the DESCARTES code) and then the creamery supplies that milk to residents in other locations (called a grocery in the DESCARTES code). This technique models the movement of milk products from farms to consumers. Consumption of locally produced leafy vegetables and milk products is also supported.

The DESCARTES and CiderF codes require external data. These data include releases from facilities, atmospheric transport, production and distribution of milk and leafy vegetables,

growing season dates, animal diets, and information regarding human diets and lifestyle activities. These external data sets and their use with the environmental accumulation and dose codes are summarized below.

Air Transport Data – The atmospheric transport code, HYSPLIT provides daily integrated radionuclide air concentrations and surface deposition rates. These data are used as input to the DESCARTES and CiderF codes: the deposition rates and air concentrations are used in the DESCARTES code; the air concentrations are passed on for use in the CiderF code. The meteorological data used by the HYSPLIT code to estimate the air concentrations and deposition rates are not addressed in this report; however, the regional data are applied repeatedly to obtain a distribution of potential concentrations and deposition rates.

Facility Release Data – In this application setting, the HYSPLIT code is used to model transport of unit releases of ^{131I}I from a number of facilities. Because the concentration equations are linear, estimates of historical releases from the facilities can be used to scale the transport results to obtain air concentrations and deposition rates. The time history of release data for every releasing facility are required inputs to the scaling utility code.

Animal Feeding Regimes – Radionuclide concentrations in animal products (cow and goat milk, meat, poultry, and eggs) are estimated as the product of the ingested activity and an animal to food product transfer factor. The equations in DESCARTES are based on the concept of feeding regimes to account for the various types of feeds consumed by livestock. A utility code develops the daily ingestion rates of the various types of vegetation consumed by animals, based on generalized feeding regime data. These daily ingestion rates are then stored in data files. These data sets are accessed during a given run of the DESCARTES computer code and a realistic animal diet is randomly selected for each location and season.

Milk and Leafy Vegetable Production and Distribution – Data about the commercial production and distribution of milk and leafy vegetables within the project domain are used in DESCARTES and CiderF. Much of this information is collected in data files accessed by DESCARTES. These data have been published for dose reconstruction activities for Mayak (Mokrov et al. 2007).

Human Dietary Data – Human diets are an important factor in determining doses from airborne ^{131}I. The food types modeled in DESCARTES and CiderF are leafy vegetables, other vegetables, fruit, grain, fresh dairy products, stored dairy products, meat, poultry and eggs. Dietary information and recommended lifestyle and exposure information for Mayak is provided in (Rovny et al. 2009). This information is used to produce reference diets used in CiderF.

4.1 Environmental Accumulation (DESCARTES) Equations

The primary equations solved by the DESCARTES code are provided in this section. The equations were developed for the HEDR project (Snyder et al. 1994) and are further described in Eslinger and Napier (2019). The equations are solved on a daily basis because the DESCARTES code supports daily, weekly or monthly outputs. Descriptions of the parameters in the equations are given further below.

The biomass rate of change over time (January 1 – June 30) is provided in the following differential equation:

$$\frac{dB}{dt}=\frac{k_g}{2}\left[1-\cos\left(\frac{2\pi t}{t_{tot}}\right)\right]B\left[\frac{B_{\max}-B}{B_{\max}}\right] \tag{DES-1}$$

The biomass rate of change over time with senescence (July 1 – December 31) is provided in the following differential equation:

$$\frac{dB}{dt}=\frac{k_g}{2}\left[1-\cos\left(\frac{2\pi t}{t_{tot}}\right)\right]B\left[\frac{B^*_{\max}-B}{B^*_{\max}}\right]-k_s\left(B-B_{\min}\right) \tag{DES-2}$$

The maximum biomass adjusted for senescence is provided in the following equation:

$$B^*_{\max}=\frac{k_g\left(B_{\max}\right)^2}{B_{\max}\left(k_g-k_s\right)+k_sB_{\min}} \tag{DES-3}$$

The foliar interception fraction is provided in the following equation:

$$f_v=1-e^{-\alpha B} \tag{DES-4}$$

The translocation rate constant is provided in the following equation:

$$\lambda_{trans}=\lambda_{weath}\left(\frac{f_{trans}}{1-f_{trans}}\right) \tag{DES-5}$$

The upper soil layer activity rate of change is provided in the following differential equation:

$$\frac{dQ_{usl}}{dt}=f_sI-Q_{usl}\left(\lambda_{perc}+\lambda_{rad}+\lambda_{splash}\right)+Q_{ov}\lambda_{weath}-R_{resus}+R_{senc,iv}+R_{senc,ov} \tag{DES-6}$$

The root zone activity rate of change is provided in the following differential equation:

$$\frac{dQ_{rz}}{dt}=Q_{usl}\lambda_{perc}-Q_{rz}\left(\lambda_{leach}+\lambda_{rad}\right)-R_{root} \tag{DES-7}$$

The outer vegetation activity rate of change is provided in the following differential equation:

$$\frac{dQ_{ov}}{dt}=f_vI-Q_{ov}\left(\lambda_{weath}+\lambda_{rad}+\lambda_{trans}\right)+Q_{usl}\lambda_{splash}-R_{senc,ov}+R_{resus} \tag{DES-8}$$

The inner vegetation activity rate of change is provided in the following differential equation:

$$\frac{dQ_{iv}}{dt}=Q_{ov}\lambda_{trans}-Q_{iv}\lambda_{rad}+R_{root}-R_{senc,iv} \tag{DES-9}$$

The deposition rate of resuspended upper soil layer material is provided in the following equation:

$$R_{resus} = \frac{V_d Q_{usl} ML}{\rho_{usl}} \quad \text{(DES-10)}$$

The rate of inner vegetation senescence (July 1 – December 31) is provided in the following equation:

$$R_{senc,iv} = \frac{Q_{iv}}{B} k_s \left(B - B_{\min}\right) \quad \text{(DES-11)}$$

The rate of outer vegetation senescence (July 1 – December 31) is provided in the following equation:

$$R_{senc,ov} = \frac{Q_{ov}}{B} k_s \left(B - B_{\min}\right) \quad \text{(DES-12)}$$

The rate of uptake through roots (January 1 – June 30) is provided in the following equation:

$$R_{root} = Q_{rz} \frac{CR}{\rho_{rz}} \left(\frac{dB}{dt}\right) \quad \text{(DES-13)}$$

The rate of uptake through roots (July 1 – December 31) with senescence is provided in the following equation:

$$R_{root} = Q_{rz} \frac{CR}{\rho_{rz}} \left[\frac{dB}{dt} + k_s \left(B - B_{\min}\right)\right] \quad \text{(DES-14)}$$

The quantity to concentration conversion for other vegetables, grain, pasture, alfalfa and silage (subscript p) and leafy vegetables and fruit (subscripts p,iv and p,ov) is provided in the following equation:

$$C_p = \frac{Q_{iv} + Q_{ov}}{B} \quad C_{p,iv} = \frac{Q_{iv}}{B} \quad C_{p,ov} = \frac{Q_{ov}}{B} \quad \text{(DES-15)}$$

The quantity of nuclide consumed by an animal at location l and time t from N_f food crops is provided in the following equation:

$$A_{cons}(t,l) = \sum_{f=1}^{N_f} R_f \, C_f(t - th_s, l)\, e^{-\lambda_{rad} th_s} \quad \text{(DES-16)}$$

The animal product concentration at location l and day t is provided in the following equation:

$$C_{ap}(t,l) = TF_{ap} \left\{ A_{cons}(t,l) + FS_a \left[\frac{f_{usl} Q_{usl}(t,l)}{\rho_{usl}} + \frac{f_{rz} Q_{rz}(t,l)}{\rho_{rz}}\right] + I\left[M + \frac{S}{1000}\right]\right\} \quad \text{(DES-17)}$$

The undecayed concentration in commercially available creamery milk at creamery X and grocery milk at location l for day t is provided in the following equations:

$$C_{cream,X}(t) = \sum_{l=1}^{L(X)} f_{cream,X}(t,l) \sum_{r=1}^{4} f_r(t,l) C_r(t,l)$$

$$C_{groc}(t,l) = \left[\sum_{x=1}^{X(l)} f_{groc,X}(t,l) C_{cream,X}(t) \right] + f_u(t,l) C_u(t,l) + f_{other}(t,l) C_{other}(t,l) \quad \text{(DES-18)}$$

The undecayed concentration in inner (subscript iv) and outer (subscript ov) compartments of commercially available leafy vegetables at location l for day t is provided in the following equations:

$$C_{comlv,iv}(t,l) = \sum_{m=1}^{M(l)} f_{lv}(l,m) C_{lv,iv}(t,m)$$

$$C_{comlv,ov}(t,l) = \sum_{m=1}^{M(l)} f_{lv}(l,m) C_{lv,ov}(t,m) \quad \text{(DES-19)}$$

4.2 Individual Dose (CiderF) Equations

The primary equations solved by the CiderF code are provided in this section. Some food products in the ingestion dose equations have both inner and outer compartments. This subdivision supports differentiation in dose for some products that can be consumed whole, or peeled and eaten, such as tree fruit. In addition, the model explicitly accounts for the lifestyle activities of a fetus, nursing baby, and a pregnant or lactating woman. Equations CID-1 through CID-5 apply to children and adults while equations CID-6 and CID-7 apply to the special case of a fetus or nursing baby. Doses to infants include the gestational period.

The dose equations are a function of time, where time is measured in days. Thus, the dose for a specific exposure period, such as a year, is calculated by summing the daily doses for the year. The symbol t denotes the day index for the dose while $t\text{-}th_p$ denotes the day index a food was produced that is consumed on day t. This computational approach supports epidemiological studies by allowing a real person to enter or exit the modeled region on specific dates. Although many of the parameters, such as dose factors or consumption rates, are a function of the age of the individual, the age dependency is not explicitly identified in the equations.

The air immersion dose equation is as follows:

$$D_{imm}(t,l) = DF_{imm}\, \chi(t,l) \left[f_{time} + (1 - f_{time}) Sh1 \right] / 86{,}400 \quad \text{(CID-1)}$$

The groundshine dose equation is as follows:

$$D_{grd}(t,l) = \left[Q_{usl}(t,l)\, DF_{usl} + Q_{rz}(t,l)\, DF_{rz} \right] \left[f_{time} + (1 - f_{time}) Sh1 \right] \quad \text{(CID-2)}$$

The inhalation dose equation is as follows:

$$D_{inh}(t,l) = DF_{inh} \left[\frac{\chi(t,l)}{86{,}400} + Q_{usl}(t,l) \frac{ML}{\rho_{usl}} \right] BR \left[f_{time} + \left(1 - f_{time}\right) R_{io} \right] \quad \text{(CID-3)}$$

The equations for ingestion dose for all foods with inner and outer compartments concentrations accounted for in combination such as other vegetables and grains (subscript veg1)

or existing only as a single concentration compartment for animal products such as meat, milk and eggs (subscript ap) are as follows:

$$D_{ing,veg1}(t,l) = DF_{ing} \sum_{p} C_p(t - th_p, l)\, R_p\, f_d\, e^{-\lambda_{rad} th_p}$$
$$D_{ing,ap}(t,l) = DF_{ing} \sum_{ap} C_{ap}(t - th_p, l)\, R_p\, e^{-\lambda_{rad} th_p} \quad \text{(CID-4)}$$

The equation for ingestion dose from crops with inner and outer vegetation compartments concentrations accounted for separately (leafy vegetables and fruit) is as follows:

$$D_{ing,veg2}(t,l) = DF_{ing} \sum_{p} \left[C_{p,iv}(t - th_p, l) + C_{p,ov}(t - th_p, l)\, L_{proc} \right] R_p\, f_d\, e^{-\lambda_{rad} th_p} \quad \text{(CID-5)}$$

The equations for the inhalation dose to a fetus or nursing baby are as follows:

$$D_{inh,fetus}(t,l) = DF_{pre} A_{inh,mother}(t,l)$$
$$D_{inh,baby}(t,l) = DF_{inh} A_{inh,baby}(t,l) + DF_{nurs} A_{inh,mother}(t,l) \quad \text{(CID -6)}$$
$$where\ A_{inh,x}(t,l) = BR\left[Q_{usl}(t,l)\left(\frac{ML}{\rho_{usl}}\right) + \frac{\chi(t,l)}{86{,}400} \right]\left[f_{time} + (1 - f_{time}) R_{io} \right]$$

The equations for the ingestion dose to fetus or nursing baby are as follows:

$$D_{ing,fetus}(t,l) = DF_{pre} A_{ing,mother}(t,l)$$
$$D_{ing,baby}(t,l) = DF_{ing} A_{ing,baby}(t,l) + DF_{nurs} A_{ing,mother}(t,l) \quad \text{(CID -7)}$$
$$\text{where } A_{ing,x}(t,l) = \left\{ \begin{array}{l} \sum_{p} R_p\, f_d\, C_p(t - th_p, l) e^{-\lambda_{rad} th_p} + \\ \sum_{p} R_p\, f_d \left[C_{p,iv}(t - th_p, l) + C_{p,ov}(t - th_p, l)\, L_{proc} \right] e^{-\lambda_{rad} th_p} + \\ \sum_{ap} R_p\, f_d\, C_{ap}(t - th_p, l) e^{-\lambda_{rad} th_p} \end{array} \right\}$$

4.3. Definition of Parameters for DESCARTES and CiderF Equations

The parameters used in the equations presented above listed alphabetically and defined in Table 4.1. The descriptions all assume that release quantities are measured in Bq.

Table 4.1 Definition of Terms in the Iodine Environmental Accumulation and Dose Equations

Term	Definition
86,400	Conversion factor, s d^{-1}
α	Empirical foliar interception constant m^2/kg(dry)
λ_{leach}	Leaching rate from root zone to deep soil, d^{-1}
λ_{perc}	Percolation rate from upper soil layer to root zone, d^{-1}
λ_{rad}	Radiological decay constant, d^{-1}
λ_{splash}	Rainsplash rate constant, d^{-1}
λ_{trans}	Plant translocation rate, d^{-1}
λ_{weath}	Weathering rate, d^{-1}
ρ_{usl}	Upper soil layer areal density to a depth of 1 mm, kg(wet) m^{-2}
ρ_{rz}	Root zone soil areal density to a depth of 15 cm, kg(wet) m^{-2}
$\chi(t,l)$	Integrated daily radionuclide air concentration on day *t* at location *l*, Bq s m^{-3} d^{-1}
$A_{cons}(t,l)$	Animal radionuclide consumption rate on day *t* at location *l*, Bq d^{-1}
$A_{ing,x}(t,l)$	Radionuclide intake rate on day *t* via ingestion for individual *x* at location *l*, where *x* = nursing baby or lactating mother, Bq
$A_{inh,x}(t,l)$	Radionuclide intake rate on day *t* via inhalation for individual *x* at location *l*, where *x* = nursing baby, or lactating mother, Bq
B	Current daily biomass, kg(dry) m^{-2}
B^{*}_{max}	Maximum biomass adjustment factor, kg(dry) m^{-2}
B_{max}	Maximum potential biomass, kg(dry) m^{-2}
B_{min}	Minimum (winter) biomass, kg(dry) m^{-2}
BR	Breathing rate, m^3 d^{-1}
$C_{ap}(t-th_p,l)$	Animal product radionuclide concentration at time of harvest at location *l*, where *ap* = milk, beef, chicken, eggs, Bq L^{-1} (milk) or Bq $[kg\ wet]^{-1}$ (others)
$C_p(t-th_p,l)$	Radionuclide concentration in food or feed crop at time of harvest, where *p* = other vegetables, grain, pasture, alfalfa, and silage at time $t-th_p$ past harvest at location *l*, Bq $[kg(dry)]^{-1}$
$C_{p,iv}(t-th_p,l)$	Radionuclide concentration in the inner vegetation compartment at time of harvest, where *p* = leafy vegetables or fruit at location *l*, Bq $[kg(dry)]^{-1}$
$C_{p,ov}(t-th_p,l)$	Radionuclide concentration in the outer vegetation compartment at time of harvest, where *p* = leafy vegetables or fruit at location *l*, Bq $[kg(dry)]^{-1}$
CR	Ratio of the radionuclide concentration in a unit mass of vegetation to the radionuclide concentration in a unit mass of soil, Bq $[kg_{vegetation}(dry)]^{-1}$ per Bq $[kg_{soil}(wet)]^{-1}$
$C_v(h_v,l)$	Animal feed radionuclide concentration harvested on date *h* at location *l*, where *v* denotes grain, pasture, grass hay, alfalfa, manger hay and silage, Bq $[kg(dry)]^{-1}$
DF_{imm}	Immersion dose rate factor, $Gy_{thyroid}$ d^{-1} per Bq m^{-3} or Sv d^{-1} per Bq m^{-3}
DF_{ing}	Ingestion dose factor, $Gy_{thyroid}$ $[Bq_{ingested}]^{-1}$ or Sv $[Bq_{ingested}]^{-1}$
DF_{inh}	Inhalation dose factor, $Gy_{thyroid}$ $[Bq_{inhaled}]^{-1}$ or Sv $[Bq_{inhaled}]^{-1}$
DF_{nurs}	Dose factor relating the dose to the nursing baby to intake of the mother, $Gy_{thyroid,baby}$ $[Bq_{intake,mother}]^{-1}$
DF_{pre}	Dose factor relating the dose to the fetus (prenatal) to intake of the mother, $Gy_{thyroid,fetus}$ $[Bq_{intake,mother}]^{-1}$
DF_{rz}	Dose rate factor for radionuclides in the root zone soil, $Gy_{thyroid}$ d^{-1} per Bq m^{-2} or Sv d^{-1} per Bq m^{-2}
DF_{usl}	Dose rate factor for radionuclides in the upper soil layer or surface activity, $Gy_{thyroid}$ d^{-1} per Bq m^{-2} or Sv d^{-1} per Bq m^{-2}
$D_{grd}(t,l)$	Dose from groundshine on day *t* at location *l*, $Gy_{thyroid}$ or Sv
$D_{imm}(t,l)$	Air immersion dose on day *t* at location *l*, $Gy_{thyroid}$ or Sv
$D_{ing,ap}(t,l)$	Ingestion dose from animal product *ap* on day *t* at location *l*, where *ap* = beef, poultry, eggs, or milk, $Gy_{thyroid}$ or Sv
$D_{ing,veg1}(t,l)$	Ingestion dose from local food crops with a single compartment on day *t* at location *l*, $Gy_{thyroid}$ or Sv
$D_{ing,veg2}(t,l)$	Ingestion dose from local food crops with two compartments on day *t* at location *l*, $Gy_{thyroid}$ or Sv

Term	Definition
$D_{ing,x}(t,l)$	Ingestion dose to individual x at location l on day t during the exposure period, where x = fetus or nursing baby, $Gy_{thyroid}$ or Sv
$D_{inh}(t,l)$	Inhalation dose on day t at location l, $Gy_{thyroid}$ or Sv
$D_{inh,x}(t,l)$	Inhalation dose to individual x at location l during the exposure period, where x = fetus or nursing baby, $Gy_{thyroid}$ or Sv
f_d	Food specific dry-weight to wet-weight conversion factor, kg(dry) $[kg(wet)]^{-1}$
f_{rz}	Fraction of root zone soil consumed by an animal in a day, equal to $1-f_{usl}$, dimensionless
f_s	Soil deposition fraction, equal to $1-f_v$, dimensionless
FS_a	Animal soil ingestion rate, where a denotes chicken, cattle or goat, kg(wet) d^{-1}
f_{time}	Fraction of days spent outdoors, dimensionless
f_{trans}	Fraction of outer vegetation deposition that translocated to the inner vegetation compartment, dimensionless
f_{usl}	Fraction of soil in the upper soil layer consumed by an animal in a day, equal to $1-f_{rz}$, dimensionless
f_v	Vegetation foliar interception fraction, dimensionless
h_v	Julian day on which feed type v was most recently harvested
I	Areal deposition rate, Bq m^{-2} d^{-1}
k_g	Growth rate constant, d^{-1}
k_s	Senescence rate constant, d^{-1}
l	Location of interest
L_{proc}	Food-processing retention fraction, dimensionless
M	Surface area of cattle manger directly exposed to atmospheric deposition, m^2
ML	Mass-loading factor for local soil in air, kg m^{-3}
Q_{iv}	Activity in the inner vegetation compartment, Bq m^{-2}
Q_{ov}	Activity in the outer vegetation compartment, Bq m^{-2}
Q_{rz}	Activity in the root zone soil layer, Bq m^{-2}
Q_{usl}	Activity in the upper soil layer, Bq m^{-2}
R_{io}	Ratio of indoor air to outdoor air activity, dimensionless
R_p	Food-product consumption rate for food crop or animal product, p, kg(wet) d^{-1} for all foods except milk and L d^{-1} for milk
R_{resus}	Rate of radionuclide redeposition on vegetation from resuspension of soil, Bq m^{-2} d^{-1}
$R_{resus,iv}$	Rate of radionuclide transfer from the inner vegetation compartment of plants to the soil by vegetable senescence, Bq m^{-2} d^{-1}
$R_{resus,ov}$	Rate of radionuclide transfer from the outer vegetation compartment of plants to the soil by vegetable senescence, Bq m^{-2} d^{-1}
R_{root}	Rate of radionuclide uptake through roots, Bq m^{-2} d^{-1}
R_{v_a}	Quantity of feed type v that animal a consumes in a day, kg(dry) d^{-1}
S	Stock tank dilution factor, m^{-1}
$Sh1$	Shielding factor for semi-infinite plumes, dimensionless
t	Day of interest
TF_{ap}	Animal product transfer factor, where ap denotes milk from a goat, milk from a cow, beef, poultry, or eggs, d L^{-1} for milk or d $kg(wet)^{-1}$ for beef, poultry or eggs
th_p	Holdup time from collection or harvest to consumption, where p is a food crop or animal product, d
th_s	Holdup time for stored feed crops, d
t_{tot}	Total number of days in the year being evaluated, 365 or 366 (leap years)
V_d	Local deposition velocity of resuspended soil back to soil or vegetation, m d^{-1}

4.4 Release Source Terms

Monthly releases of ^{131}I are available (Mokrov et al. 2008a), on pages 23-24 for the D and DB radiochemical plants. Annual releases from the reactors are provided in Table 4.1 of the same report. Some release data for 1968 through 1972 can be derived from the stack monitoring values of the same report. The typical use of CiderF is to calculate annual or lifetime doses, so using monthly source terms from the largest releases should not cause a significant loss of information.

Deterministic release estimates use the best estimate monthly values, implemented as constant daily releases for each day in the month. If published monthly values are not available, then the annual values are divided into equal releases for each day. Stochastic releases are implemented as a scaling factor (triangular distribution on [minimum, best estimate, maximum]) for the annual releases. The minimum and maximum ends of the triangular distribution are also from Mokrov et al. (2008a).

Source entries are made for every month a facility operated. Preparation of the release keywords is performed in a spreadsheet and then the keywords are copied and pasted into the text keyword files. The spreadsheet contains a separate sheet for every release facility. A utility code generates monthly and annual releases from every facility. The monthly releases for each realization are scaled to match the annual release for that realization.

Best estimate values for the monthly releases (Ci) by facility are provided in

Figure 4.1. As expected, releases from the radiochemical processing plants B and DB dominate the releases. The best estimate annual releases summed across all active facilities are provided in Figure 4.2.

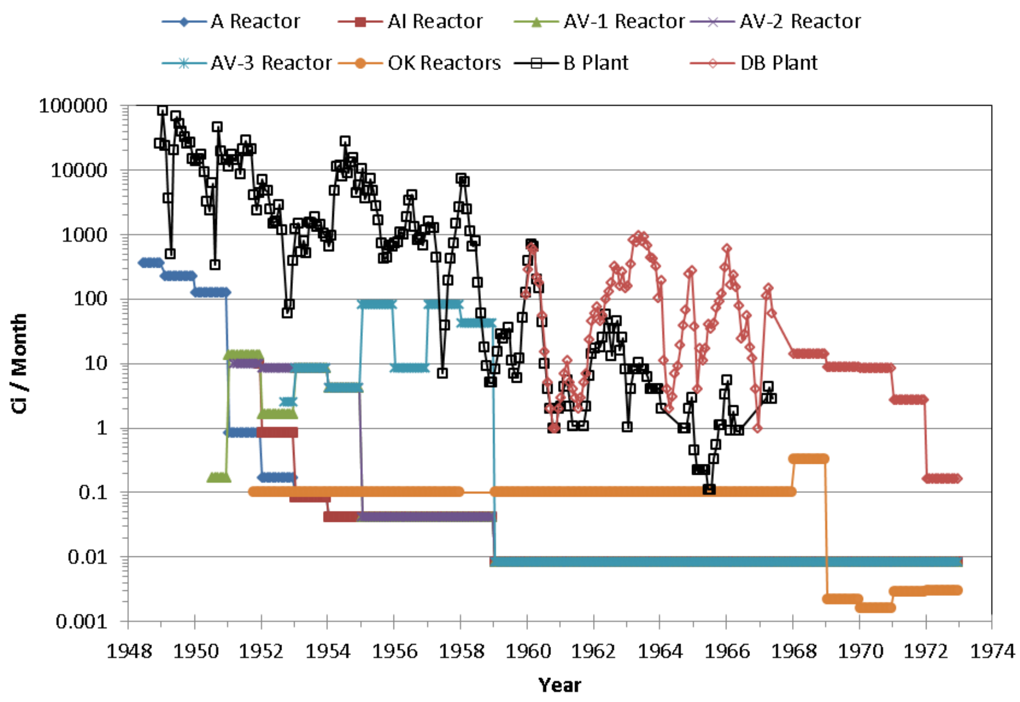

Figure 4.1 Monthly Best Estimate Atmospheric Releases of ${}^{131}I$ by Facility

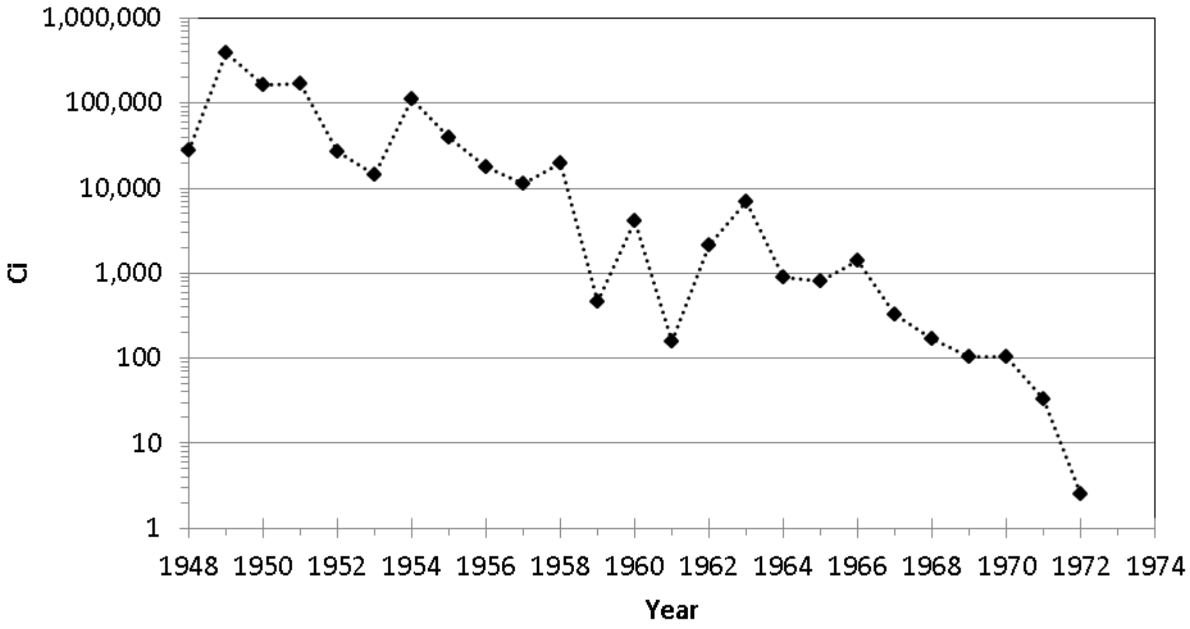

Figure 4.2 Annual Best Estimate Atmospheric Releases of ${}^{131}I$

Summary statistics on the annual releases of ^{131}I (TBq) to the air by facility for the years 1948 through 1972 are provided in Table 4.2. The summary was developed from the 1500-realization stochastic case.

4.5. Iodine Speciation

The speciation algorithm for ^{131}I is taken from (Napier et al. 2008). The algorithm has two steps. First, the fraction of ^{131}I in each form is sampled uniformly from within its specific range. Second, the three fractions are normalized so the total equals one. The fractions of each form are defined as follows:

- elemental iodine, uniformly distributed on (0.10, 0.45),
- organic iodine, uniformly distributed on (0.20, 0.35), and
- particulate iodine, uniformly distributed on (0.20, 0.65).

Speciation for the best-estimate run uses 0.44 for particulate form, 0.29 for the organic form, and 0.27 for the elemental form. These values are the median values from the set of 1500 speciation fractions, rounded to two decimals. Values for the particulate form ranged from 0.219 to 0.671 with a standard deviation of 0.093. Values for the organic form ranged from 0.168 to 0.505 with a standard deviation of 0.063. Values for elemental form ranged from 0.102 to 0.499 with a standard deviation of 0.087.

Table 4.2 Estimated annual releases (TBq) of 131I to the atmosphere for 1948 through 1972. The summary statistics are derived from a 1500-realization stochastic analysis. The release quantities are rounded to 3 digits of accuracy for presentation purposes.

Year	Best Estimate	Minimum Release	Median Release	Maximum Release	Standard Deviation
1948	1040	757	1040	1300	109
1949	14600	10500	14600	18500	1660
1950	6070	4360	6040	7660	687
1951	6200	4440	6170	7830	708
1952	1000	717	998	1260	114
1953	534	386	531	671	59.7
1954	4180	2990	4160	5280	477
1955	1450	1040	1450	1820	162
1956	666	478	663	841	75.7
1957	411	303	409	512	43.0
1958	736	535	732	923	82.1
1959	17.4	12.9	17.3	21.2	1.59
1960	153	116	152	188	12.5
1961	5.87	4.50	5.85	7.14	0.530
1962	78.6	57.2	78.1	97.6	7.80
1963	253.	182	252	318	28.6
1964	32.6	23.6	32.5	41.0	3.70
1965	30.0	21.7	29.9	37.7	3.40
1966	51.9	37.4	51.7	65.7	5.90
1967	12.3	8.9	12.2	15.4	1.40
1968	6.30	4.58	6.27	7.91	0.70
1969	3.87	2.78	3.85	4.89	0.44
1970	3.83	2.76	3.81	4.83	0.44
1971	1.23	0.89	1.22	1.54	0.14
1972	0.093	0.071	0.092	0.113	0.008

4.6. Biosphere and Individual Parameter Uncertainty Distributions

The majority of the parameters used in the Mayak evaluation were taken from those developed for the Hanford Environmental Dose Reconstruction project. Snyder et al. (1994) provide detailed descriptions of the parameters and their distributions. For those parameters adopted unchanged from Snyder et al. (1994), the values and distribution types are listed in Table 4.3. The reader is referred to that document for more details. In Table 4.4, for normal and lognormal distributions, the “min” and ‘max” values listed are the 1% and 99% truncation limits of the applied distributions.

Table 4.3. Definition of Terms in the Environmental Accumulation and Dose Equations

Para-meter	Description of parameter	Units	Estimates			Type of* distribution
			Central	min	max	
α	Empirical foliar interception constant	m^2/kg (dry weight)	–	1.0	4.0	U
λ_{leach}	Leaching rate of ^{131}I from root zone to deeper soil layers	day^{-1}	–	$4.0 \cdot 10^{-6}$	$5.0 \cdot 10^{-3}$	U
λ_{perc}	Percolation rate of ^{131}I from upper soil layer to root zone	day^{-1}	–	0.14	0.914	U
λ_{rad}	^{131}I radioactive decay constant	day^{-1}	$8.625 \cdot 10^{-2}$	–	–	Constant
λ_{weath}	rate of ^{131}I vegetation surface activity decrease due to weathering	day^{-1}	0.0495	0.0347	0.0866	T
ρ_{rz}	Average areal density of root soil layer with a depth of 0.1 to 15 cm	kg/m^2 (wet weight)	–	186	230	U
ρ_{usl}	Average areal density of upper soil layer with depth of 0.1 cm	kg/m^2 (wet weight)	–	1.10	1.45	U
BR	Daily age dependent breathing rates:	m^3/day				T
	(0-0.5) years		1.62	0.5	4.9	
	(0.5-2.0) years		5.14	1.7	15.4	
	(2.0-7.0) years		8.71	2.9	26.1	
	(7.0-12.0) years		15.3	5.1	45.9	
	(12.0-17.0) years		17.7	5.9	53.9	
	17 years and over		22.0	7.3	66.0	Triangular
B_{max}	Leafy Vegetables	kg(dry) m^{-2}	0.2	0.07	0.6	Triangular
	Other Vegetables		0.5	0.17	1.2	Triangular
	Tree Fruit		0.54	0.3	2.0	Triangular
	Grain		0.14	0.09	0.3	Triangular
	Pasture		0.3	0.1	0.7	Triangular
	Grass Hay		0.3	0.1	0.6	Triangular
	Alfalfa		0.2	0.07	0.4	Triangular
	Silage		0.3	0.1	0.6	Triangular
B_{min}	Leafy Vegetables	kg(dry) m^{-2}	0.01			Constant
	Other Vegetables		0.01			Constant
	Tree Fruit		0.27			Constant
	Grain		0.01			Constant
	Pasture		0.04			Constant
	Grass Hay		0.03			Constant
	Alfalfa		0.01			Constant
	Silage		0.01			Constant

Parameter	Description of parameter	Units	Estimates			Type of* distribution
			Central	min	max	
CR	^{131}I plant-to-soil concentration ratio	$\left(\frac{Ci/kg(dry)}{Ci/kg(dry)}\right)$	–	0.01	0.25	LU
DF_{imm}	Age-dependent immersion dose rate factor (relating to semi-infinite ^{131}I cloud)	–				U
	for absorbed dose to the thyroid	$\left(\frac{rad/day}{Ci/m^3}\right)$	–	$2.8\cdot10^3$	$1.1\cdot10^4$	
DF_{ing}	Ingestion age- and gender dependent dose factor	–				LN
	for absorbed dose to the thyroid for:	rad/Ci	–			
	(0-0.5) years		$1.4\cdot10^7$	$2.2\cdot10^6$	$5.5\cdot10^7$	
	(0.5-2.0) years		$1.3\cdot10^7$	$2.0\cdot10^6$	$5.1\cdot10^7$	
	(2.0-7.0) years		$7.8\cdot10^6$	$1.2\cdot10^6$	$3.1\cdot10^7$	
	(7.0-12.0) years		$4.1\cdot10^6$	$6.4\cdot10^5$	$1.6\cdot10^7$	
	(12.0-17.0) years		$2.5\cdot10^6$	$3.9\cdot10^5$	$9.9\cdot10^6$	
	17 years and over (male)		$1.4\cdot10^6$	$2.2\cdot10^5$	$5.5\cdot10^6$	
	17 years and over (female)		$1.7\cdot10^6$	$2.7\cdot10^5$	$6.7\cdot10^6$	
DF_{inh}	Inhalation age- and gender dependent dose factor	–	–	–	–	LN
	for absorbed dose to the thyroid	rad/Ci	$8.5\cdot10^6$	$1.3\cdot10^6$	$3.3\cdot10^7$	
	(0-0.5) years					
	(0.5-2.0) years		$8.1\cdot10^6$	$1.3\cdot10^6$	$3.2\cdot10^7$	
	(2.0-7.0) years		$4.8\cdot10^6$	$7.5\cdot10^5$	$1.9\cdot10^7$	
	(7.0-12.0) years		$2.4\cdot10^6$	$3.8\cdot10^5$	$9.5\cdot10^7$	
	(12.0-17.0) years		$1.5\cdot10^6$	$2.3\cdot10^5$	$5.9\cdot10^6$	
	17 years and over (male)		$1.0\cdot10^6$	$1.6\cdot10^5$	$3.9\cdot10^6$	
	17 years and over (female)		$1.2\cdot10^6$	$1.9\cdot10^5$	$4.7\cdot10^6$	
DF_{nurs}	Absorbed dose to the nursing baby's thyroid (rad) from the intake of ^{131}I by the mother (Ci)	rad/Ci	$2.4\cdot10^6$	$3.3\cdot10^5$	$1.0\cdot10^7$	LN
DF_{pre}	Dose factor relating the absorbed dose to the fetus (i.e. prenatal) from intakes to the mother from ICRP Publication 88	rad/Ci	$1.6\cdot10^6$	$2.5\cdot10^5$	$6.3\cdot10^6$	LN
DF_{rz}	Dose rate factor for ^{131}I in the soil root zone [2]	$\left(\frac{rem/day}{Ci/m^2}\right)$	–	49	88	U

Para-meter	Description of parameter	Units	Estimates			Type of* distribution
			Central	min	max	
DF_{usl}	Dose rate factor for upper soil layer or surface activity [2]	–	–	–	–	U
	– for absorbed dose to the thyroid	$\left(\frac{rad / day}{Ci / m^2}\right)$	–	84	120	
f_d	Dimensionless dry-weight to wet-weight conversion factor:	$\frac{kg\ (\text{dry})}{kg\,(\text{wet})}$	–	–	–	U
	leafy vegetables		–	0.05	0.09	
	other vegetables		–	0.04	0.26	
	fruit trees		–	0.13	0.35	
	grains		–	0.85	1.00	
FS_a	Daily soil ingestion rate for a chicken	kg/day (initial weight)	–	$6.0{\cdot}10^{-5}$	$1.2{\cdot}10^{-2}$	U
FS_a	Daily soil ingestion rate for cow (bull) at various durations of stay on pasture lands :	–	–	–	–	T
	24 h/d – all pasture	kg/day (initial weight)	0.5	0.25	1.0	
	12 h/d – half		1.0	0.50	1.5	
	0 h/d – no pasture		2.0	1.0	4.0	
f_{time}	Fraction of day spent outdoors:	–	–	–	–	T
	1. Children (male/female) from 0 to 2 years:		–	–	–	
	winter		0.0	0.0	0.13	
	spring		0.04	0.0	0.17	
	summer		0.13	0.0	0.29	
	autumn		0.04	0.0	0.17	
	2. Children (male/female) from 2 to 17 years (urban):	–	–	–	–	T
	winter		0.10 / 0.05	0.04 / 0.04	0.13 / 0.13	
	spring		0.13 / 0.08	0.04 / 0.04	0.17 / 0.17	
	summer		0.35 / 0.22	0.08 / 0.08	0.38 / 0.38	
	autumn		0.13 / 0.08	0.04 / 0.04	0.17 / 0.17	
	3. The same for rural population:	–	–	–	–	
	winter		0.13 / 0.08	0.04 / 0.04	0.17 / 0.17	
	spring		0.21 / 0.17	0.04 / 0.04	0.23 / 0.23	
	summer		0.34 / 0.32	0.13 / 0.13	0.50 / 0.50	
	autumn		0.21 / 0.08	0.04 / 0.04	0.23 / 0.23	
f_{time}	4. Adults in urban areas (17 years and over):	–	–	–	–	T
	winter		0.05 / 0.07	0.0 / 0.0	0.17 / 0.17	
	spring		0.18 / 0.29	0.0 / 0.0	0.31 / 0.31	

Para-meter	Description of parameter	Units	Estimates			Type of* distribution
			Central	min	max	
	summer		0.22 / 0.29	0.04 / 0.04	0.41 / 0.41	
	autumn		0.10 / 0.15	0.0 / 0.0	0.31 / 0.31	
	5. Adults of rural areas (17 years and over):	–	–	–	–	
	winter		0.33 / 0.21	0.04 / 0.04	0.37 / 0.37	
	spring		0.44 / 0.36	0.04 / 0.04	0.50 / 0.50	
	summer		0.47 / 0.29	0.06 / 0.06	0.50 / 0.50	
	autumn		0.34 / 0.21	0.04 / 0.04	0.37 / 0.37	
f_{trans}	Fraction of outer vegetation deposition that translocates to the inner vegetation compartment	–	–	0.01	0.2	LU
k_g	Rate constant for biomass: Leafy Vegetables	day^{-1}	0.11	–	–	Constant
	Other Vegetables		0.09	–	–	
	Tree Fruit		0.09	–	–	
	Grain		0.12	–	–	
	Pasture		0.12	–	–	
	Grass Hay		0.12	–	–	
	Alfalfa		0.27	–	–	
	Silage		0.12	–	–	
k_s	Parameter taking into account inner and outer vegetation senescence processes: Leafy Vegetables	day^{-1}	0.07	–	–	Constant
	Other Vegetables		0.08	–	–	
	Tree Fruit		0.07	–	–	
	Grain		0.08	–	–	
	Pasture		0.09	–	–	
	Grass Hay		0.09	–	–	
	Alfalfa		0.15	–	–	
	Silage		0.09	–	–	
L_{proc}	Food processing retention fraction	–	–	0.15	0.75	U
ML	Mass loading factor for local soil in air	kg/m^3 (dry weight)	$7.0\cdot10^{-8}$	$1.4\cdot10^{-8}$	$3.5\cdot10^{-7}$	LN
R_{io}	Indoor to outdoor ^{131}I volumetric activities ratio	–	–	0.35	1.0	U
S	Stock tank dilution factor	m^{-1}	1.0	0.2	1.6	T
$Sh1$	Shielding factor for semi-infinite plumes (depending on wall properties)	–		0.05	0.93	U
TF_{ap}	Animal product transfer factor (specific activity of	$\left(\frac{Ci/kg}{Ci/day}\right)$	–	0.004	0.054	U

Parameter	Description of parameter	Units	Estimates			Type of* distribution
			Central	min	max	
	^{131}I in beef (Ci/kg) per daily intake of ^{131}I to the cow's organism (Ci/day)					
TF_{ap}	The same for chicken eggs (wet weight)	$\left(\frac{Ci/kg}{Ci/day}\right)$	–	1.5	6.0	U
TF_{ap}	The same for goat's milk (Ci/l)	$\left(\frac{Ci/l}{Ci/day}\right)$	0.27	0.04	1.15	LN
TF_{ap}	The same for cow's milk (public herd cows)	$\left(\frac{Ci/l}{Ci/day}\right)$	0.012	0.0073	0.016	N
TF_{ap}	The same for cow's milk (private cows)	$\left(\frac{Ci/l}{Ci/day}\right)$	$9.2\cdot10^{-3}$	$1.6\cdot10^{-3}$	$5.2\cdot10^{-2}$	LN
TF_{ap}	The same for chicken meat	$\left(\frac{Ci/kg}{Ci/day}\right)$	–	$4.0\cdot10^{-3}$	$9.4\cdot10^{-2}$	U
th_p	Holdup time for stored feed crops:	–	–	–	–	U
	all kinds of vegetables, fruits, cereals	day	–	0.0	7.0	
	milk from private cows or goats		–	0.0	2.0	
	purchased (commercial) milk		–	1.0	4.0	
	stored milk products (sour cream, butter, etc.)		–	14.0	60.0	
	meat (beef)		–	7.0	21.0	
	poultry		–	2.0	10.0	
	eggs		–	0	21	
V_d	Local deposition velocity of resuspended soil back to soil or vegetation $\frac{Ci/m^2}{Ci\cdot s/m^3}\cdot\frac{s}{day}$	m/day	–	86.4	$2.59\cdot10^{3}$	U

* *U – uniform; LU – lognormally uniform; N – normal; PU – piecewise-uniform; T – triangular; LN – lognormal.*

The only parameter revised for the Mayak releases in Table 4.4 to date is the dose factor relating dose to prenatal thyroid to intake by the mother, DF_{pre}. This distribution has been updated using the newer value from ICRP Publication 88 and assigned a GSD of 2.0 as derived for the other dose factors in Snyder et al. (1994).

In the calculations performed to date for the Techa and EURT, the rural parameters of Table 4.4 have been used. The indoor/outdoor fractions are similar to, but slightly different from, the values used for the Techa external parameter T_2. Future recalculations should adopt the values of T_2.

4.7. Environmental Accumulation Modelling

A suite of preprocessors are used to prepare data sets for the DESCARTES environmental accumulation code for best estimate or stochastic data sets. The following two major cases were run in DESCARTES:

- Best Estimate Case: All DESCARTES parameters were set to best estimate values and the best estimate case from the dispersion code is used (1 realization).
- Stochastic Case: A full set of stochastic inputs (1500 realizations) is used in DESCARTES and the stochastic case from the dispersion code.

4.7.1 Frost Date Libraries

The concept of first and last frost dates is used to determine the annual growing season. A separate library file containing frost dates is required for each model year when computing plant-related concentrations. These files are produced by the utility code FrostpUno. FrostpUno is a modification of the FROSTP code (Miley et al. 1994) that applies the same definition of frost dates for all locations in the dose model domain and multiple years. The code writes a file of randomized frost dates for every model year.

Historical weather information for Balandino Airport, Chelyabinsk, Russian Federation is available on the web (WeatherSpark 2012). Reading from the interactive graph, reproduced here as static Figure 4.3, for averages on the "dashboard" at that website, one can obtain the following range of dates. Last spring frost (overnight low reached down to 32 °F): average day is April 12 (Julian day 102); 10% are as early as March 30 (Julian day 89); 10% are as late as May 2 (Julian day 122). First spring frost (overnight low reached down to 32 °F): average day is October 24 (Julian day 297); 10% are as early as October 2 (Julian day 275); 10% are as late as November 10 (Julian day 314).

The best estimate run uses a spring frost date of April 12 (Julian day 102) and a fall frost date of October 24 (Julian day 297). These dates are applied to all dose model locations. The same dates are used for every year.

The stochastic runs use uniform distributions for the frost dates. The spring date is uniform on the range (89,122) and the fall date is uniform on the range (275,314). These same stochastic definitions are used at all locations for every year.

A suite of library files containing temperatures that trigger changes between animal feeding season dates is required when computing animal product concentrations. Generally, these library files are produced by the utility code FROSTP. General feeding season dates are available (Mokrov et al. 2007b), section 3 and more information is provided in Appendix B of (Rovny et al. 2009). However, little or no date information is available for any specific year. Thus, the frost dates described in Section **Ошибка! Источник ссылки не найден.**are used to trigger feeding season changes.

We do note that average monthly temperatures for the Argayash weather station are available (Table A.3.3 of (Drozhko and Khokhryakov 2003)) for 1949 through 1972. Even though we didn't take this approach, the April and October data could be used to shift the overall average dates to account for cooler and warmer months than the overall average. If this approach were used, one would probably reduce the stochastic ranges to about a week on either side of the shifted average.

4.7.2 Animal Diet Libraries

A series of library files containing daily animal diet information is required for computing concentrations of animal products in DESCARTES. The library files were generated using the utility program ADIETP. The ADIETP code reads a keyword file and outputs a randomized library containing daily diet information. Although the animal feeding regimes are allowed to change from year to year, this analysis uses the same feeding regime every year. General animal consumption information is taken from (Mokrov et al. 2008b), sections 3 and 4. Additional information is provided in Appendix A of Rovny et al. (2009). The distributions are summarized in Tables 4.4 and 4.5 below.

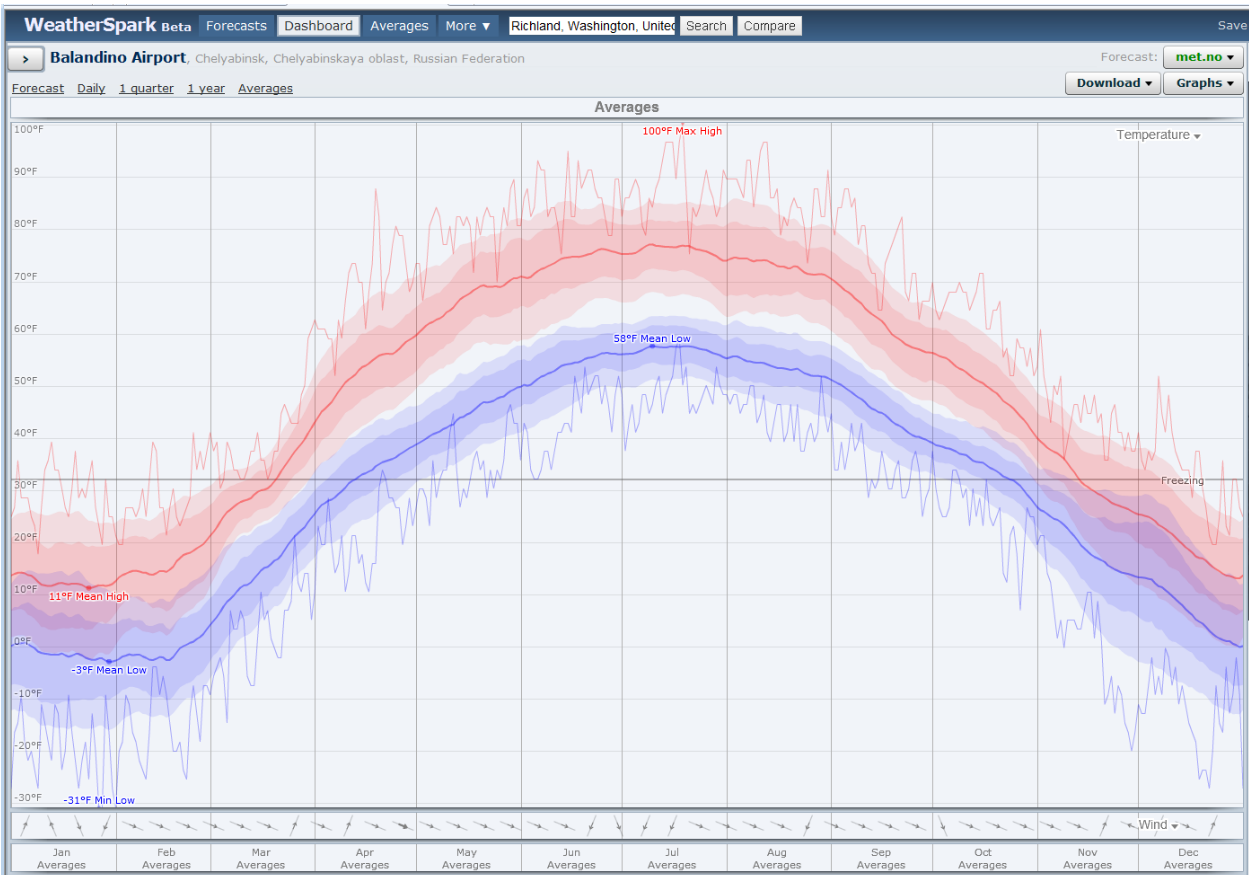

Figure 4.3. Historical temperature data for Balandino Airport, Chelyabinsk, Russian Federation

Table 4.4. Parameters of feed consumption for cows and goats (from 1949 to 1963)

Parameter	Designation, units	Cow	Goat
Daily consumption of herbage (fresh weight)	M_{grass}, kg/day	50<br>(45 – 60)	6.5<br>(5 – 8)
Daily consumption of pasture soil	M_{soil}, kg/day	1.2<br>(0.5 – 1.8)	0.06<br>(0.05 – 0.07)
Daily consumption of hay during stabling period (dry weight)	M_{hay}, kg/day	10<br>(8 – 12)	1,7<br>(1.3 – 2.0)
Daily consumption of water	M_{wates}, l/day	50	10
Fraction of time spent by an animal in open area during a grazing season:	$k_{outdoor}$, h/day		
– public herd;		24	–
– private herd.		16	16 – 18

Table 4.5 Values of water and food consumption for chickens

Parameter	Designation, units	Mean consumption (range)
Average daily consumption of fresh plants during summer period	M_{food}, kg/day	0.130<br>(0.120 – 0.150)
Average daily consumption of soil with feed	M_{soil}, kg/day	0.003<br>(0.002 – 0.004)
Average daily consumption of water without regard for feed at keeping in a pen during summer period	M_{water}, l/day	0.65<br>(0.5 – 0.8)

4.7.3 Commercial Leafy Vegetable and Milk Distribution

The dose calculation system has the capability to provide concentrations of radioiodine in foods produced in one location and shipped to other locations. For the calculations performed to date for the Southern Urals populations, it has been assumed that all individuals consume locally produced foods. This is a reasonable assumption for most villages other than Ozersk, for which it is known that collective farms supplied the bulk of fresh foods. However, because individual-specific information is not available for the subjects, a simple assumption has been used of local food sources, and these capabilities have not been applied.

4.7.4 Runs of the DESCARTES Code

Two runs of the DESCARTES code were made; best estimate and stochastic. Each of these runs produces a suite of 27 “media” files containing concentrations in a variety of media to be used in the CiderF code.

4.7.5 Individual Diets

Because individual-specific information is not available for the subjects, a simple assumption has been used of local food sources and rural diets for all calculations. Age- and sex-dependent dietary information was provided by Mokrov et al. (2008b). This reference did not supply explicit uncertainty ranges on the dietary information; where this was lacking a generic default of plus or minus 10% in a uniform distribution was used. It is important to note that this basic information is also used indirectly in the Techa River and EURT ingestion estimates. The central values are presented in Table 4.6. The default uncertainty distribution is uniform with lower and upper bounds at 10 percent above or below the central value.

Table 4.6. Annual consumption rates (kg/yr) of locally produced foods for various age and sex combinations

**Consumer Group**	fmilk	smilk	lveg	oveg	fruit	grain	eggs	beef	poultry
Child, 0 to 6 months	14.0	2.5	0.0	0.0	0.0	0.0	0.0	0.0	0.0
Child, >6 months to 2 year	108.6	19.2	2.3	11.3	31.0	18.3	0.0	29.2	1.5
Child, rural, >2 years to 7 years	155.1	27.4	7.3	22.6	4.0	32.9	0.0	29.2	1.5
Child, urban, >2 years to 7 years	155.1	27.4	7.3	22.6	3.7	32.9	0.0	29.2	1.5
Child, rural, >7 years to 12 years	155.1	27.4	14.6	73.0	4.0	146.0	0.0	36.5	1.8
Child, urban, >7 years to 12 years	155.1	27.4	14.6	73.0	3.7	146.0	0.0	36.5	1.8
Child, rural, >12 years to 17 years	155.1	27.4	25.6	73.0	4.0	171.6	0.0	47.5	2.4
Child, urban, >12 years to 17 years	155.1	27.4	25.6	73.0	3.7	171.6	0.0	47.5	2.4
Adult, rural	155.1	27.4	36.5	94.9	8.0	186.2	14.6	69.4	3.5
Adult, urban	155.1	27.4	36.5	94.9	7.3	186.2	13.0	69.4	3.5
Suckling child, 0 to 6 months	0.0	0.0	0.0	0.0	0.0	0.0	0.0	0.0	0.0
Suckling child, >6 months to 2 year	108.6	19.2	2.3	11.3	31.0	18.3	0.0	14.6	0.7
Adult, rural, pregnant/nursing female	170.6	30.1	30.1	30.1	8.8	204.8	16.1	76.3	3.8
Adult, urban, pregnant/nursing female	170.6	30.1	30.1	30.1	8.0	204.8	14.3	76.3	3.8

5.0 Doses from medical treatment

Individual members of the Techa River Cohort were brought to the URCRM clinic for examinations; many of these examinations included either radiography or fluorography exposures. Individual medical exposure histories for individuals examined at the URCRM clinic are presented in blocks by examined individual; including date of specific examinations, the type of x-ray apparatus that was used (in historical order –URD, DIA, TUR, RUM), indicators of the type of examination and target organ and orientation of the patient (lateral, posterior, anterior), and the number of frames (for radiography) or viewings (for fluoroscopy). In essence, x-ray doses are added to the individual's appropriate annual organ dose summary at the proper time.

The dose per radiographic exam or per minute of fluoroscopy, as a function of the type of x-ray apparatus, has been pre-calculated using software developed for the purpose at the St. Petersburg Institute of Radiation Hygiene (Degteva et al. 2005; 2007). These are for a reference person; real individuals vary substantially about the reference value, and there is additional uncertainty in the basic unit doses themselves. For all types of radiography and fluorography procedures, a shared uncertainty with a normal distribution with mean of 1 and standard deviation of 0.25, truncated at the lower value of 0.1, is assigned. An additional unshared uncertainty multiplier described by a normal distribution with mean of 1 and standard deviation of 0.1 is used.

Fluoroscopy examinations are characterized by a lengthy exposure necessary for visual evaluation. The duration of fluoroscopy examinations was not entered in the URCRM archives. Therefore, the values for this important parameter can be taken from literature sources or from expert evaluations only. The duration of x-raying taken during individual fluoroscopy procedures can vary from 0.6 to 9 minutes for examination of chest organs, from 3 to 7 minutes for complex examination of esophagus, stomach and duodenum, and from 2 to 6 minutes for examination of colon. These values are used as unshared uncertainty ranges; triangular distributions are used with modes set at the midpoints of the ranges.

For some individual records, the number of radiography frames are not recorded. To estimate the number of frames taken the distribution of the number of retakes in those who do have records was reviewed, and a simple algorithm developed to estimate the number of frames randomly. That algorithm is to take the least integer value of a function of the form

$\mathrm{INT}(4 - 3 * \sqrt{(1-r)})$, where r is a random number selected uniformly between 0 and 1.

This distribution gives about 55% one exposure, 33% two exposures, and 12% three exposures.

Note that the St. Petersburg database only includes potential doses to 12 organs, while the remainder of the dosimetry system accounts for 23 organs. This is believed to be sufficient for assessing the potential for confounding of the risk assessments by medical exposure. Current plans are to include medical exposures for all members of the SUPER Cohort for whom there are medical records at URCRM.

6.0 Summary of Techa River, EURT, and radioiodine parameter uncertainty distributions

As noted in Section 4, the bulk of the uncertainty distributions used for the atmospheric iodine analyses are taken directly from the Hanford Environmental Dose Reconstruction Project, from whence the codes were taken. The only exceptions are the source term, climatic (frost date), animal feeding quantities, and reference age-dependent food ingestion quantities, which were defined in JCCRER Project 1.4.

The parameters used in the Techa River, EURT, and Medical Exposure calculations are summarized in Table 6.1, excerpted from Napier et al. (2018). The designation of each parameter as shared or unshared and either Berkson or Classical is given in Table 6.1. The shared parameters are used as input to the first TRDS-MC module; the unshared parameters are used as inputs to the individual exposure module.

The Monte Carlo procedures used in the TRDS-MC code require that at least as many realizations be performed as there are parameters input. Because the outer loop of shared parameters includes a total of 1455 individual parameters, when duplicates by radionuclide, location, and time period are counted, the code is run for 1500 realizations. The individual dose code 'only' has a total of 476 parameters, so may be run only for 500 realizations; but because this does not ensure complete coverage of the 1500 outer-loop realizations, the total 1500 realizations are used for each individual dose estimate.

Planned Improvements in parameter uncertainty distributions:

As detailed in the preceding sections, work continues to reduce uncertainties and to improve the description of uncertainties in the Techa River Dosimetry System. In addition to ongoing work on individual tracing and residence history verification, the following parameters are being evaluated:

• A_0 Future calculations will use a slightly broadened unshared uncertainty with a multiplier with a normal distribution with mean of 1.0 and standard deviation of 0.16

• $D_{Riv,L,y}$ For downriver distances of less than 200 km, a shared multiplier with a normal distribution with mean of 1.0 and standard deviation of 0.4, truncated at 0.1, will be used. For downriver distances greater than 200 km, a lognormal distribution with mean of 1.0 and GSD of 3.31 will be employed

• T_1 The time by the river as a function of age will be revised to acknowledge that some people did not spend time by the riverbank. This will involve a convolution of the probability of river visits with the distribution of time spent when visiting.

• T_3 The importance of revisiting and harmonizing the external dose components of the atmospheric iodine pathway with the assumptions used for the river external exposures will be evaluated and perhaps included in future atmospheric radioiodine pathway calculations

• $D_{Sr,y}$ The evolution of EURT fallout radionuclides vertical migration into the soil column over time will be investigated

• DF_{Sr} Ongoing work will define the inter-individual variability of the strontium internal dose factors. The results of the ongoing strontium bone dosimetry will be included in future calculations for the entire SUPER Cohort

• f_L^{Sr-90} The village-to-village differences in radionuclide uptake with river water will continue to evolve as more whole-body counts are made on cohort members, because these are used within the calculational system to define the uncertainty distributions of intake for unmeasured residents of each village.

The future versions of TRDS will be used to calculate doses for the extended cohort – the Southern Urals Populations Exposed to Radiation (SUPER), that will comprise all people who resided in areas contaminated by discharges of liquid radioactive materials into the Techa River and by the accidental releases from the Kyshtym Accident, as well as thyroid doses from the atmospheric releases of ^{131}I, including those who were exposed in-utero. Medical exposures of those with records at URCRM will also be estimated for use in evaluation of potential cofounding of the risk estimates.

Table 6.1. Summary table of TRDS parameters and their uncertainty structure. Distributions are shown in terms of parameter multipliers as follows: Norm[mean, st. dev]; LogNorm[geometric mean, geometric st. dev]; Uniform[min/mean-max/mean]; LogUniform[min/mean-max/mean]; Custom – empirical distribution

**Parameter**	**Description**	**Multiplicative uncertainty effect distribution**				**Data source of uncertainty estimates**
		**Unshared (stochastic)**		**Shared (lack of knowledge)**		
		**shape**	**Number of parameters per individual and realization**	**shape**	**Number of parameters per individual and realization**	
**Common to internal and external exposure**						
$G^{90Sr}(L_i(y))$	Surface deposition of ^{90}Sr (Bq m^{-2}) at location *L* from fallout from the EURT	*Norm*[1,0.2]	Limit on moves set to 20 → 20 parameters	*Norm*[1,0.05]	84 villages →84 parameters	Shared – URCRM database<br>Unshared – expert estimate
**Internal exposure**						
$I_i^{T,r}(L_i(y), \tau_i(y))$	Individual, *i*, intake from the Techa River (Bq) (function of age *τ* at year *y*)					
I_y^{90Sr}	Annual ^{90}Sr intake for adults of the reference settlements (reference ^{90}Sr intake)	*Norm*[1,0.25]	53 time slice values → 53 parameters	Withing village or household Custom	42 settlements → 42 parameters	Shared – from measurements of ^{90}Sr body-burden<br>Unshared – from measurement errors
$f_{L_i(y)}^{90Sr}$	Ratio of ^{90}Sr intake for location (*L*) to reference ^{90}Sr intake	Considered in I_y^{90Sr}				Shared – from village-specific measurements of ^{90}Sr body-burden if no individual
$\xi_i = \begin{cases} 1 \\ IMR_i \\ HSR_i \end{cases}$	An individual modifier depending on availability and uncertainty of individual to model ratio (*IMR*) of ^{90}Sr in the body or an	Considered in I_y^{90Sr}				IMR_i – unshared<br>HSR_i – shared withing household

	average for household specific *IMRs* (*HSR*)					
$\alpha^{^{90}Sr}(\tau_i(y))$	Annual ^{90}Sr intake for children relative to that for adults	*Norm*[1,0.2]	Apply to one age to one year; after age 10, equal to adult → 11 parameters	within age group *Norm*[1,0.1]	Apply to one age to one year; after age 10, equal to adult → 11 parameters	Shared – the error of the intake age dependence<br>Unshared – expert estimate
$R^{r:\,^{90}Sr}_{L_i(y)}$	Annual-average location and time-specific ratio of radionuclide r -to-^{90}Sr intake			within village<br>*LogNorm*[1,2]<br>*Uniform*[0.5-1]	11 nuclides × 42 villages → 462 parameters	Combined due to: global fallouts and radionuclide transport model [12]
$I_i^{E,r}$	Annual radionuclide-specific intake function per unit of ^{90}Sr surface deposition in EURT	*LogNorm*[1,2]	Limit on moves set to 20 → 20 parameters	within village<br>*LogNorm*[1,3]	6 nuclides x 84 villages → 504 parameters	URCRM database<br>Shared – from errors of village-specific contamination<br>Unshared – from studies of dietary intakes
$E_r(y, \tau_i(y))$	Conversion factor for ^{90}Sr surface deposition (Bq m^{-2}) to annual intake (Bq) for radioisotope *r*. Depend on age at time of intake and year after deposition.	Considered in $I_i^{E,r}$				Shared – from errors of age-dependence of dietary intakes
$DF_{ro}(t-y_0, \tau_i(y_0))$	Conversion factor (Gy Bq^{-1}) for dose accumulated in organ o in year $Y-y$ from intake of radionuclide r (function of age at intake)	^{90}Sr - *LogNorm*[1,1.25]<br>Other radionuclides - *LogNorm*[1,2]	11 nuclides x 23 organs → 253 parameters	within age with autocorrelation *Norm*[1,0.1]	11 nuclides x 23 organs → 253 parameters	Shared – expert estimate<br>Unshared – based on individual variability of skeleton mass (for ^{90}Sr) or whole-body mass from literature

						data and URCRM database
**External exposure**						
$A_o(\tau_i(y))$	Conversion factor from absorbed dose in air to absorbed dose in organ *o* (function of age at time of exposure)	*Uniform*[0.9-1.1]	1	within age with autocorrelation *Uniform*[0.9-1.1]	23 organs → 23 parameters	Expert estimates
$D_{Riv}\big(y, L_i(y)\big)$	Annual absorbed dose in air on the Techa River shoreline at location *L* (Gy year^{-1})			within village with autocorrelation *Norm*[1,0.1]	42 villages → 42 parameters	Expert estimates
$D^{\ ^{90}Sr}(\mathrm{L}(y))$	Normalized dose rate in air outdoors in time *y* (Gy year^{-1} per Bq m^{-2}) from EURT fallout			within village *Uniform*[0.9,1.1]	15 month → 15 parameters	Expert estimates
$R_{\frac{out}{Riv}}\big(L_i(y)\big)$	Bank to residence ratio (function of distance of individual's home from river)	*LogUniform*[min-max]- village specific parameters	42 villages → 42 parameters			Information about village-specific locations of households
$R_{\frac{out}{in}}\big(L_i(y)\big)$	Indoor/Outdoor ratio (function of building type)	*Uniform*[0.28, 1.72]	Limit on moves set to 20 → 20 parameters			Expert estimates
$T_1(\tau_i(y)), T_2(\tau_i(y))\ and\ T_3(\tau_i(y))$	Time fraction (annual-average) spent on the riverbank, outdoors and indoors, respectively	*LogNorm*[1, 2.7]	T_1 and T_3 are considered as independent → 2 parameters			Surveys-based estimates and expert estimates

ACKNOWLEDGEMENTS

This work was funded by the U.S. Department of Energy's Russian Health Studies Program and the Federal Medical Biological Agency of the Russian Federation under the auspices of the Joint Coordinating Committee for Radiation Effects Research

REFERENCES

Annenkov BN, Dibobes IK, Alexakhin RM (eds). Radiobiology and radioecology of agricultural animals. Moscow, Atomizdat, 1973 (in Russian).

Avramenko MI, Averin AN, Drozhko EG, Glagolenko YuV, Loboiko BG, Mokrov YuG, Romanov GN, Kotov ES, Filin VP (1997) Accident of 1957 and East Urals Radioactive Trace. Radiat Saf Probl (Mayak Prod Assoc Sci J) 3:18–28 (in Russian)

Balonov MI, Bruk GY, Golikov VY, Barkovsky AN, Kravtsova EM, Kravtosova OS, Mubasarov AA, Shutov VN, Travnikova IG, Howard BJ, Brown JE, Strand P. Assessment of current exposure of the population living in the Techa River basin from radioactive releases of the Mayak facility. Health Phys. 92(2):134-147; 2007.

Borovinskikh PG, Borchaninova KI, Gukalova NM, Ivanov VA, Marey AN, Saurov MM, Severin SF, Khrustalev VP, Yartsev EI. A study of sanitary situation in area of Techa, Iset, Tobol Rivers connected with stopping of releases of Plant No. 817. Moscow: Biophysics Institute; Technical Report; 1958 (in Russian).

Borovinskikh PG, Dubrovina ZV, Panteleev LI, Rasin IM, Serebryakova AA. A problem of 90Sr elimination rate in the human organism. Chelyabinsk: Urals Research Center for Radiation Medicine; Technical Report 1340; 1963 (in Russian).

Brokhovich VB. The chemical plant "Mayak." History, the streamer of events. Ozersk: Mayak Production Association; 1996 (in Russian).

Degteva MO, Shagina NB, Tolstykh EI, Shishkina EA, Kozyreva OV, Napier BA. Calculations of individual dose from environmental exposures on the Techa River and EURT using TRDS2016 for members of the TRC. Urals Research Center for Radiation Medicine and Pacific Northwest National Laboratory; Final Report for Milestone 13, Part 1. April 2017a.

Degteva MO, Shagina NB,Vorobiova MI, Shishkina EA, Peremyslova LM, Tokareva EE, Anspaugh LR, Napier BA. Individualization and validation of external doses for individuals who lived in the upper Techa River villages. Chelyabinsk and Salt Lake City: Urals Research Center for Radiation Medicine and University of Utah; Final Report for Milestone 3; April 2011.

Degteva MO, Shishkina EA, Volchkova AYu, Bougrov NG, Napier BA. Uncertainties in parameters for external dose calculation in the Techa River dosimetry systemTRDS-2016MC. Urals Research Center for Radiation Medicine and Pacific Northwest National Laboratory; Addendum to Milestone Report 11 Part 1. September 2016.

Degteva MO, Tolstykh EI, Shishkina EA, Shagina NB, Kozyreva OV, Napier BA. Calculations of individual dose in red bone marrow from environmental exposures for members of the Techa River Cohort. Urals Research Center for Radiation Medicine and Pacific Northwest National Laboratory; Final Report for Milestone 11/13, Part 2. October 2017b.

Degteva, MO, Anspaugh LR, Napier BA, Tolstykh EI, Shagina NB, Kozheurov VP, Vorobiova MI, Tokareva EE, Shishkina EA. Analysis of the main factors contributing to uncertainty in internal dose from 90Sr and feasibility evaluation for reduction in uncertainty. Chelyabinsk: Urals Research Center for Radiation Medicine; Final Report for Milestone 8; 1999.

Degteva MO, Golikov VYu, Vorobiova MI, Barkovsky AN, Zubkova TI, Kozyreva OV, Anspaugh LR, Napier BA. Development of a Protocol for the Reconstruction of Individual Medical Doses for Members of the Extended Techa River Cohort. Chelyabinsk and Salt Lake City: Urals Research Center for Radiation Medicine and University of Utah; Final Report for Milestone 17; September 2005.

Degteva MO, Shagina NB, Vorobiova MI, Golikov VYu, Barkovsky AN, Kozyrev AV, Anspaugh LR, Napier BA. Reconstruction Of Individual Medical Doses For Members Of The Extended Techa River Cohort. Chelyabinsk and Salt Lake City: Urals Research Center for Radiation Medicine and University of Utah; Final Report for Milestone 19; December 2007.

Draxler RR, Stunder B, Rolph G, Stein A, Taylor A. HYSPLIT4 User's Guide, Version 4, Air Resources Laboratory, National Oceanic and Atmospheric Administration (NOAA). Silver Spring, Maryland. 2013.

Eslinger, PW, KS Lessor, and SJ Ouderkirk. User Instructions for the CIDER Dose Code, PNWD2252 HEDR, Battelle, Pacific Northwest Laboratories, Richland, Washington. 1994.

Eslinger PW, Napier BA. User Instructions for the CiderF Individual Dose Code and Associated Utility Codes, PNNL-22699, Pacific Northwest National Laboratory, Richland, Washington, 2013a. https://www.pnnl.gov/main/publications/external/technical_reports/PNNL-22699.pdf

Eslinger PW, Napier BA. Analysis Approach and Data Package for Mayak Public Doses, PNNL22704, Pacific Northwest National Laboratory, Richland, Washington, 2013b. https://www.pnnl.gov/main/publications/external/technical.../PNNL-22704.pdf

ICRP 67. International Commission on Radiological Protection, Age-dependent Dose to Members of the Public from Intake of Radionuclides: Part 2: Ingestion Dose Coefficients. Publication 67. Annals of the ICRP, 23 (3/4), Pergamon Press, Oxford, 1993.

Izrael YA (ed) Atlas of the East Ural and Karachay radioactive trace including forecast up to 2047—Moscow, IGCE Roshydromet and RAS. 2013. http://downloads.igce.ru/publications/Atlas/ CD_VURS/about.html. Accessed 4 July 2016

Izrael YA, Artemov EM, Vasilenko VN, Nazarov IN, Nakhutin AI, Uspin AA, Kyamkin AM Radioactive contamination of Urals region by Mayak Production Association. In: Radioactivity under nuclear explosions and accidents. Proceedings of International

Conference, Moscow, 24–26 April, 2000. Gidrometeoizdat, St. Petersburg, pp 411–424 , 2000 (in Russian).

JNREG Joint Norwegian-Russian Expert Group for Investigation of Radioactive Contamination in the Northern Areas. Sources Contributing to Radioactive Contamination of the Techa River and Areas Surrounding the "Mayak" Production Association, Urals, Russia. Norwegian Radiation Protection Authority, Østerås, Norway , 1997.

Khokhryakov VV, Drozhko EG, Romanov GN, Mokrov YuG, Kyamkin AM, Volobuev PV, Vorobiova MI, Kostychenko VA, Akleyev AV. Characteristics of anthropogenic radiation impact on territory and population. In: Consequences of an anthropogenic radiation incident and rehabilitation problems of the Urals Region. Comtehprint Publisher, Moscow, pp 8–77, 2002 (in Russian).

Khokhryakov VF, Erokhin RA, Plotnikova LA. Estimation of bone doses for residents of Settlement "M." Ozersk: Southern Urals Biophysics Institute; technical report; 1968 (in Russian).

Korsakov Yu, Bronnikov V, Borchikov A. Radioactive contamination of flood-plain soils of the Techa River (from Muslyumovo village to mouth). Ozersk: Mayak PA' Technical Report; 1970 (in Russian).

Kostyuchenko VA, Peremyslova LM, Popova IY, Kazachyonok NN, Melnikov VS. Main dependencies in reduction of radiation exposure to the population of the Southern Urals. Health Phys 103(1):42–46, 2012.

Kozheurov VP, Degteva MO. Dietary intake evaluation and dosimetric modeling for the Techa River residents based on in vivo measurements of strontium-90 in teeth and skeleton. Sci Total Environ 142:63–72; 1994.

Kozheurov VP, Zalyapin VI, Shagina NB, Tokareva EE, Degteva MO, Tolstykh EI, Anspaugh LR, Napier BA. Statistical analysis of individual dosimetric data and the evaluation of uncertainties in instrumental techniques used for 90Sr-body-burden evaluation (whole-body count and tooth-beta count). Chelyabinsk and Salt Lake City: Urals Research Center for Radiation Medicine and University of Utah; Final Report for Milestone 1; 2000.

Kravtsova EM, Kolotygina NV, Barkovsky AN. External irradiation of the residents of the Muslyumovo village of Chelyabinsk Oblast. In: Chukanov VN, ed. Radiation, Ecology, Health. Part II: Impact of radiation on the public health. Ekaterinburg; 1994:13–16 (in Russian).

Lebedev VM, Zaitsev YuA, Andrianov YuA, Nikolaenko LA, Ivina YuV, Kossenko MM, Nazarov AV, Yakovleva VP, Evtushenko NN, Savostin VA, Akleyev AV, Rait MK. Development and organization of computerized database of dosimetric, demographic and medical observations of population exposed to uranium fission products. Chelyabinsk: Urals Research Center for Radiation Medicine; URCRM Technical Report No. 1548, Parts 1 and 2; 1985 (in Russian).

Marey A.N., Ivanov V.A., Saurov M.M., Alekseeva O.G., Afanasiev G.G., Babayan R.S., Baranova B.O., Borovinskikh P.G., Vdovina V.A., Vergilesova O.S., Zhakov Yu.A.,

Ivanova K.D., Karpova A.P., Kolbina N.S., Kolosova E., Korneeva T.D., Kutepova N.T., Laricheva L.P., Lebedev N.D., Lebedeva G.M., Markova L., Mareninova T.A., Minina E.M., Nikiforova V.A., Nikolaev Yu.M., Pashnina V.I., Pentiugov V.N., Prikhod'ko E.I., Plotnikov M.F., Pushina L.N., Pyatkova T.M., Severin S.F., Semin A.S., Serebryakova A.N., Springin V.N., Stepanova M.Ya., Chernikova L.K., Shmelev L.V., Yartsev E.I. Study of the sanitary-hygienic situation and the state of health of the population in the area contaminated with liquid radioactive waste from the facility N 817. Annual report. Moscow, 1961, 348 p.

Marey AN, Ilyin DI, Kardeeva AA, Lukacher GYa, Liperovskaya ES, Djachenko VN, Gubin VA, Istomina AG, Merkulova TN, Korzukhina NA, Golubitskaya NN, Gorelov II. Impact of Mendeleev's plant wastes released into the Techa River on the sanitary conditions and population health in coastal villages. Moscow: Institute of Biophysics; Technical Report; 1952 (in Russian).

Marey AN, Saurov MM, Zhakov YuA, Sipko GM. Fission product contents in diets of population of contaminated area in the period 1950 –1964. Moscow: Biophysics Institute; Technical Report; 1966 (in Russian).

Maslyuk A.I. Distribution of a population according to the time of contact with contaminated water reservoir during its use for household needs. Bull Radiat Medicine 3: 119-122; 1980 (in Russian).

Miley TB, Eslinger PW, Nichols WE, Lessor KS, Ouderkirk SJ. User Instructions for the DESCARTES Environmental Accumulation Code, PNWD-2251 HEDR, Battelle. Pacific Northwest Laboratories, Richland, Washington. 1994. http://dx.doi.org/10.2172/10154088.

Mokrov YG, Martyushov VZ, Stukalov PM, Ivanov IA, Levunina ES, 2007. Milestone 8: Production and Delivery of Food to Ozersk, Reconstruction of Dose to the Residents of Ozersk from Operation of the Mayak Production Association: 1948-2002, US-Russian Joint Coordinating Committee on Radiation Effects Research Project 1.4, Ozersk, Russia.

Mokrov YG, Lyzhkov AV, Muzrukov VA, Pyatin NP, Rovny SI, Anspaugh LR, Napier BA, 2008a. Milestone 7: Reconstruction of Atmospheric Releases of I-131 from Mayak Radiochemical Plant Stacks for the Period from 1948 to 1970, Part 2: Results of the Reconstruction of 131I Releases from the Stacks of the Reactors and Radiochemical Plants, Reconstruction of Dose to the Residents of Ozersk from Operation of the Mayak Production Association: 1948-2002, US-Russian Joint Coordinating Committee on Radiation Effects Research Project 1.4, Ozersk, Russia.

Mokrov YG, Martyushov VZ, Stukalov PM, Antonova TA, Ivanov IA, Rovny SI, Anspaugh LR, Napier BA, 2008b. Milestone 6: Changes in Population Food Ratio and Demographic Parameters for Ozersk in 1948e2002. Historical Age dependent Food-consumption Rates, Reconstruction of Dose to the Residents of Ozersk from Operation of the Mayak Production Association: 1948-2002, US-Russian Joint Coordinating Committee on Radiation Effects Research Project 1.4, Ozersk, Russia.

Molchanova I, Mikhailovskaya L, Antonov K, Pozolotina V, Antonova E. Current assessment of integrated content of long-lived radionuclides in soils of the head part of the East Ural Radioactive Trace. J Environ Radioact 138:238–248, 2014.

Müller H, Gering F, Pröhl G. Model Description of the Terrestrial Food Chain and Dose Module FDMT in RODOS PV4.0. Neuherberg, GSF, 1999.

Napier BA, Degteva MO, Tolstykh EI, Shishkina EA, Smith MA, Preston DL. Description of dose distributions with uncertainties for members of the Techa River and East Urals Radioactive Trace Cohorts. Urals Research Center for Radiation Medicine and Pacific Northwest National Laboratory; Final Report for Milestone 14. April 2018a.

Napier BA, Eslinger PW, Degteva MO, Shagina NB, Smith MA. Description of TRDS-2016MC and Atmospheric Iodine Programs: documentation and maintenance package. Urals Research Center for Radiation Medicine and Pacific Northwest National Laboratory; Final Report for Milestone 15, Part 2. October 2018b.

Napier BA, Eslinger PW, Tolstykh EI, Vorobiova MI, Tokareva EE, Akhramenko BN, Krivoschapov VA, Degteva MO. Calculations of individual doses for the TRC members exposed to atmospheric radioiodine from the Mayak releases. Urals Research Center for Radiation Medicine and Pacific Northwest National Laboratory; Final Report for Milestone 10/12; April 2015.

Napier, BA, LR Anspaugh, YG Mokrov, and SI Rovny. 2008. Milestone 4: Evaluation of Chemical Forms of 131I Involved in Atmospheric Transport, US-Russian Joint Coordinating Committee on Radiation Effects Research Project 1.4, Ozersk, Russia.

Napier BA, Shagina NB, Degteva MO, Tolstykh EI, Vorobiova MI, Anspaugh LR. "Preliminary uncertainty analysis for the doses estimated using the Techa River Dosimetry System – 2000." Health Phys 81:395−405; (2001).

Panteleev LI, Skryabin AM, Korsakov YuD, Panchenko IYa, Belova EI, Bronnikov VYa, Ivin IS, Manuylov IG, Samarina AA, Carapiltcev IA, Tabarchuk AD. Present-day radiation situation in the area of Techa River. Chelyabinsk: Urals Research Center for Radiation Medicine; Technical Report No 736; 1971 (in Russian).

Peremyslova LM. Daily diet intake of calcium and strontium-90 for children younger than one year. In: Issues of radiation hygiene, proceedings of third scientificpractical conference on radiation hygiene. Kyiv: Ministry for Public Order Protection; 1967: 133–136 (in Russian).

Romanov GN, Shejn GP, Aksenov GM. Exposure doses for the population living on East-Urals radioactive trace: current estimates. Radiat Saf Probl (Mayak Prod Assoc Sci J) 4:52–67, 1997 (in Russian).

Rovny SI, Mokrov YG, Stukalov PM, Beregich DA, Teplyakov II, Anspaugh LR, Napier BA, 2009. Methods for Calculating Thyroid Doses to the Residents of Ozersk Due to 131I Releases from the Stacks of the Mayak Production Association, Reconstruction of Dose to the Residents of Ozersk from Operation of the Mayak Production Association: 1948-2002. US-Russian Joint Coordinating Committee on Radiation Effects Research Project 1.4, PNNL-18916. Pacific Northwest National Laboratory, Richland, Washington. http://dx.doi.org/10.2172/983438.

Saurov MM. Measurement of dose-rates of external exposure and survey on life-styles of inhabitants along the Techa River. Presented at the Russian-Japanese Experts' Meeting on Epidemiological Research of Radiation Effects in the Techa River Basin in Southern Urals. January 24–28, 1992, Tokyo, Japan.

Saurov MM. Radiation-hygiene assessment of natural movement of population exposed to chronic influence of uranium fission products. Moscow: Biophysics Institute; Doctoral Thesis; 1968 (in Russian).

Sayapina R.Ya., Gusev D.I., Zikova A.S., Saurov M.M., Maslyuk A.I. Formation of a collective dose under various conditions of economic use of a radioactively polluted reservoir. Final report. Moscow, 1977, 99p.

Schwarz BS, Bolch WE. Re-evaluation of organ dose conversion factors for UF/ICRP reference computational phantoms resulting from external exposures at the Techa River due to ground contamination. Gainesville, FL: University of Florida; Report for Milestone 9 part 1; 2014.

Shagina NB, Degteva MO, Tolstykh EI, Zalyapin VI, Krivoschapov VA, Tokareva EE, Anspaugh LR, Napier BA. Algorithm for selection of the best individual estimate of internal dose on the basis of the results of calculations performed with use of three different protocols. Chelyabinsk, Salt Lake City and Richland: Urals Research Center for Radiation Medicine, University of Utah and Pacific Northwest National Laboratory; Final report for Milestone 18; April 2007.

Shagina NB, Degteva MO, Vorobiova MI, Peremyslova LM, Anspaugh LR, Napier BA. Evaluation of parameters for external dose calculation in the Techa River Dosimetry System TRDS-2012. Chelyabinsk and Salt Lake City: Urals Research Center for Radiation Medicine and University of Utah; 2012a.

Shagina NB, Vorobiova MI, Degteva MO, Peremyslova LM, Shishkina EA, Anspaugh LR, Napier BA. Reconstruction of the contamination of the Techa River in 1949-1951 as a result of releases from the "Mayak" Production Association. Radiat Environ Biophys. 51(4):349-66; 2012b.

Shagina NB, Golikov VYu, Degteva MO, Vorobiova MI, Anspaugh LR, Napier BA. Reconstruction of Individual Doses due to Medical Exposures for Members of the Techa River Cohort. Med Radiol Radiat Saf 57(3):13–25; 2012c (in Russian).

Shagina NB, Tolstykh EI, Degteva MO, Anspaugh LR, Napier BA. Age and gender specific biokinetic model for strontium in humans. J Radiol Prot 35(1):87–127, 2015.

Shishkina EA, Volchkova AYu, Degteva MO, Napier B. Dose coefficients to convert air kerma into organ dose rate values for people of different ages externally exposed to ^{137}Cs in soil. Nuclear and Radiation Safety 89(1):36-47; 2016 (in Russian).

Skryabin AM. Regularities of 90Sr behavior in food chains and human diet in the condition of global radioactive fallout. Moscow: First Medical Institute; 1971 (in Russian). Ph.D. thesis.

Skryabin AM, Safronova NG, Peremyslova LM, Polyakova EA. Hygienic assessment of the consequences of contamination of large territory by radioactive wastes. Technical report No. 1549. Chelyabinsk, Urals Research Center for Radiation Medicine, 1985 (in Russian).

Snyder SF, Farris WT, Napier BA, Ikenberry TA, Gilbert RO. 1994. Parameters Used in the Environmental Pathway and Radiological Dose Modules (DESCARTES, CIDER and CRD Codes) of the Hanford Environmental Dose Reconstruction Integrated Codes (HEDRIC). PNWD-2023 HEDR, Rev. 1, Battelle, Pacific Northwest Laboratories, Richland, Washington.

SOUL. Southern Urals Radiation Risk Research. First Annual Report on Project no. FIP6R-516478. SP3: Dosimetry for the extended Techa River cohorts. Munich: GSF; 2006.

SOUL. Southern Urals Radiation Risk Research. Midterm Report on Project no. FIP6R-516478. SP3: Dosimetry for the extended Techa River cohorts. Munich: GSF; 2007.

Stein, AF, RR Draxler, GD Rolph, BJB Stunder, MD Cohen, and F Ngan. 2015. "NOAA's Hysplit Atmospheric Transport and Dispersion Modeling System." Bulletin of the American Meteorological Society 96(12):2059-2077. doi:10.1175/BAMS-D-14-00110.1

Teverovsky EN, Ternovsky IA. Permitted levels of radioactive emissions into the atmosphere. Moscow, Energoatomizdat, 1985 (in Russian)

Tolstykh EI, Degteva MO, Peremyslova LM, Shagina NB, Shishkina EA, Krivoschapov VA, Anspaugh LR, Napier BA. Reconstruction of long-lived radionuclide intakes for Techa riverside residents: Strontium-90. Health Phys 101(1):28–47; 2011.

Tolstykh EI, Degteva MO, Peremyslova LM, Shagina NB, Vorobiova MI, Anspaugh LR, Napier BA. Reconstruction of long-lived radionuclide intakes for Techa riverside residents: ^{137}Cs. Health Phys 104(5):481–498; 2013.

Tolstykh EI, Degteva MO, Shishkina EA, Ivanov DV, Shved VA, Bayankin SN, Anspaugh LR, Napier BA, Wieser A, Jacob P. Age-dependencies of 90Sr incorporation in dental tissues: comparative analysis and interpretation of different kinds of measurements obtained for residents on the Techa River. Health Phys 85:409 – 419; 2003.

Tolstykh EI, Zalyapin VI, Shagina NB, Krivoshchapov VA, Degteva MO, Tokareva EE, Anspaugh LR, Napier BA. Estimation of individual-to-model ratios (IMR) and their uncertainty for the Techa River residents. Chelyabinsk and Salt Lake City: Urals Research Center for Radiation Medicine and University of Utah; Final report for Milestone 4; November 2002 (in Russian and English).

Tolstykh EI, Zalyapin VI, Shagina NB, Krivoshchapov VA, Degteva MO, Tokareva EE, Anspaugh LR, Napier BA. Evaluation of ETRC subcohorts with different algorithms for 90Sr body burden estimation and assessment of their uncertainties. Chelyabinsk and Salt Lake City: Urals Research Center for Radiation Medicine and University of Utah; Final report for Milestone 8; September 2003 (in Russian and English).

Tolstykh EI, Shagina NB, Peremyslova LM, Safronova NG, Degteva MO, Anspaugh LR, Napier BA. Improvement in cesium-137 intake with milk contaminated as a result of soil→

grass→ milk transfer. Chelyabinsk and Salt Lake City: Urals Research Center for Radiation Medicine and University of Utah; Unscheduled report; October 2008.

Tolstykh EI, Shagina NB, Peremyslova LM, Degteva MO, Anspaugh LR, Napier BA. Methodological Approaches to the Reconstruction of Radionuclide Intake for Residents of the East Urals Radioactive Trace and Karachay Trace. Chelyabinsk and Salt Lake City: Urals Research Center for Radiation Medicine and University of Utah; Final report for Milestones 11 and 12; March 2006.

Tolstykh EI. Peremyslova LM, ·Degteva MO, Napier BA. Reconstruction of radionuclide intakes for the residents of East Urals Radioactive Trace (1957–2011), Radiat Environ Biophys 56:27–45; 2017. DOI 10.1007/s00411-016-0677-y

Vorobiova MI, Degteva MO, Kozyrev AV, Anspaugh LR, Napier BA. External doses evaluated on the basis of the Techa River Dosimetry System approach. Chelyabinsk and Salt Lake City: Urals Research Center for Radiation Medicine and University of Utah; Final report for Milestone 6; May 1999 (in Russian and English).

Vorobiova MI, Degteva MO, Safronova NG, Akhramenko BN, Anspaugh LR, Napier BA. Methodological approaches to evaluation of parameters for external exposure of the Techa River residents. Chelyabinsk and Salt Lake City: Urals Research Center for Radiation Medicine and University of Utah; Unscheduled report; 2009.

WHO (World Health Organization. Multicentre Growth Reference Study Group). WHO Child Growth Standards: Length/height-for-age, weight-for-age, weight-for-length, weight-for-height and body mass index-for-age: Methods and development. Geneva: World Health Organization, 2006. – 312 p.
