# Supplementary material for: Dose estimates and their uncertainties for use in epidemiological studies of radiation-exposed populations in the Russian Southern Urals
Source: PLoS One. 2023 Aug 10;18(8):e0288479. doi: 10.1371/journal.pone.0288479 (PMC10414627; doi:10.1371/journal.pone.0288479)
Supplement: Attachment — Submitted filename: TRDS2016 Response to Reviewers.docx [file pone.0288479.s004.docx]

PONE-D-23-01650 review rebuttal letter

June 14, 2023

Dear Dr. Mosseau

We are pleased with the reviewers’ positive comments on our manuscript *Dose uncertainties for radiation epidemiology: research experience in the Urals region* (PONE-D-23-01650). We have now prepared a revised manuscript in which we address the reviewers’ comments and the editorial concerns raised in your decision letter. Our detailed responses to the reviewers’ comments are given below. Before that we address your editorial concerns.

Comments from the editor

1. We have modified the manuscript to follow the style described in the style requirements templates.
2. It is our understanding the *Funding Information* section of the manuscript is prepared by the journal from the *Financial Disclosure* section of the submission form. We provide an updated Financial Disclosure statement below. If more information is necessary we will provide it.
3. We would like to update/correct the funding source information for Dr. Shishkina (EAS) and the late Marina Degteva (MOD) and provide some additional information for Dr. Preston (DLP) and Mr. Napier (BAN). The revised ***Financial Disclosure*** statement should be:

***Financial Disclosure***

EAS:	Federal Medical-Biological Agency of Russia Contract N◦ 27.501.19.2 in the framework of Russian Federal Targeted Program “Provision of nuclear and radiation safety for the period 2016-2020 and for the period up to 2035”. <https://xn----btb4bfrm9d.xn--p1ai/>

and

US Department of Energy, Project Joint Coordinating Committee for Radiation Effect Research (JCCRER) dose reconstruction for the Urals. [https://www.energy.gov/ehss/international-health-studies-and-activities](https://www.energy.gov/ehss/international-health-studies-andactivities)

BAN: PNNL Contract DE-AC05-76RL01830, (US Department of Energy), Project JCCRER DOSE RECONSTRUCTION FOR THE URALS, Budget and Reporting Number HS0240030, https://www.energy.gov/ehss/international-health-studies-and-activities

DLP:	University of Southern California Prime Award # DE-HS0000091 (US Department of Energy), Project Epidemiological and Biostatistical Assistance for Project 2.2 Mayak Worker Cancer Mortality and for Project 1.2 Techa River Cohort Cancer mortality and Incidence, USC Subaward 122032572 – Mod 3, [https://keck.usc.edu](https://keck.usc.edu/) and <https://www.energy.gov/ehss/international-health-studies-and-activities>

MOD (deceased):	Federal Medical-Biological Agency of Russia Contract N◦ 27.501.19.2 in the framework of Russian Federal Targeted Program “Provision of nuclear and radiation safety for the period 2016-2020 and for the period up to 2035”. <https://xn----btb4bfrm9d.xn--p1ai/>

and

US Department of Energy, Project Joint Coordinating Committee for Radiation Effect Research (JCCRER) dose reconstruction for the Urals. [https://www.energy.gov/ehss/international-health-studies-and-activities](https://www.energy.gov/ehss/international-health-studies-andactivities)

The funders had no role in study design, data collection and analysis, decision to publish, or preparation of the manuscript.

4. The updated title for the paper is:
   Dose estimates and their uncertainties for use in epidemiological studies of radiation-exposed populations in the Russian Southern Urals.

We have revised this in the manuscript and, using the Edit Submission feature, on the online submission form.

5. An ethics statement section was added at the end of the Methods section.
6. The supporting information for this report is an extensive report on the details of the TRDS system that go beyond what is presented in this manuscript. The supporting information document (provided in a file called S1.pdf) as a whole is referred to at several places in the manuscript. However, the main text of this paper does not refer to specific locations in the supporting information.
7. The reference list was amended to include references suggested by the reviewers and to update some of the original references. The references were formatted using the PLoS style that comes with EndNote21. The formatting which appears to be consistent with the journal’s guidelines.

# Response to reviewer comments

Both reviewers provided their comments on pdf copies of the submitted file. In what follows we refer to the page numbers lines numbers provided by the manuscript management software and the page numbers in the manuscript file that we provided.

## Reviewer 1

We appreciate the reviewer’s careful reading of the manuscript and the helpful comments. As indicated below, we have made the suggested corrections and, as necessary, revised the text to address the reviewer’s concerns. We appreciate the suggested references and have cited them in the revised text.

Page 3

Lines 70- 71 : the text was modified to read “*underestimation of the risk estimate uncertainty*”

Page 4

FPSGV-HU9YN-9EH6Q-SE7LX-47HXCPage 5

Line 103: “*Techa River Dosimetry System*” is deleted and replaced by *TRDS-2016*

Line 106 :	In general, there are currently no guidelines for setting the number of realizations in a MC dosimetry system. However, in this case the number was chosen in part because of the number of parameters in the dose uncertainty model and the requirement of at least one realization per parameter for the Latin hypercube sampling algorithm used .

Line 110:	changed as suggested to *TRC* ***and*** *EURT*

Pages 6-7

Lines 123-124	Omitted the phrase *for 23 organs … as 67 years* as suggested.

Lines 144 -149	Omit bullets also drop “*while*” at beginning of line 149

Line 151	Corrected text to $I_{y}^{{}^{90}Sr}$ as in Eqn 1

Line 159	The reference to ONIS was dropped since this level of detail is of no detail almost all readers.

Pages 9 10

Lines 201-207 & 225-226	replaced *-*with “*is*” as requested

Line 226	The text was corrected to read “*is a conversion factor from*” …

Page 11

Line 236	Equation 5 was corrected as suggested

Line 239	The text was modified to read “*this parameter has the same value as for the Techa River*”

Line 240-241	“*the same as for the Techa River* “is deleted”

Line 242	We have added a short description of how the individual and population statistics were computed where the population and individual statistics are first discussed (line 309) and in the notes to Tables 2 and 3.

Line 250	“..*for both internal and external exposure*” was added as suggested

Page 12

Lines 258 and 260	reworded to be more direct along the lines suggested

Line 272 (Table 1) .The table has been modified.

Page 17

Line 294	Changed to acronyms, as suggested.

Lines 309 & 311	We have added information on the meaning of population and individual statistics here and in the notes to Tables 2 and 3..

Line 314	We have now given the median with two significant digits.

Page 18

line 327	We modified the text to indicate that the short-lived radionuclides include all radionuclides other than 137Cs.

Page 22

Lines 414 & 416	Replaced 25th percentiles with medians (18 and 174)

Page 28

Line 546+(Table 4)	We have revised the table title and added notes explaining the difference between population and individual statistics.

Page 33-34.

Line 677+ and 699	(Table 6) References and medians updated as suggested.

## Reviewer 2

We are grateful for the positive comments, the suggested corrections and references. We have revised the text to address all of the issues raised.

Page 2.

Line 24. Changed to “*Russian Southern Urals*”

Page 4.

Line 79 Changed to “… *populations of the Russian Southern Urals* …”

Line 83 And 85. The suggested references were added.

Line 84	added *(sometimes called the “Kyshtym Accident”)*
(which is somewhat ironic since the accident occurred at Mayak and Kyshtym was not really in the contaminated area)

Page 5.

Line 117. Changed to “*bone marrow*”

Page 6

Lines 135-136 modified to *Techa* ***River*** *internal exposures*

Page 9.

Line 191 modified to *Techa* ***River*** *External exposures*

Page 13.

Line 284 changed to *active* ***bone*** *marrow*

Page 14.

Line 288 Changed to *Techa River Dosimetry System (TRDS)*

Page 25

Line 452	AM changed to *active bone marrow*

Page 34

Line 695	change to *non-iodine*

Page 35

Line 752	changed to … contamination resulting from Mayak operations and fallout deposition in the EURT resulting from the 1957 Mayak waste storage tank explosion

Page 38

Lines 829 and 832 Suggested references were added
